# Mechanisms and Therapeutic Strategies for Minority Cell‐Induced Paclitaxel Resistance and Tumor Progression Mediated by Mechanical Forces

**DOI:** 10.1002/advs.202417805

**Published:** 2025-04-24

**Authors:** Xueyan Feng, Di Zhang, Guoxun Wang, Liwei Lu, Feng Feng, Xiuyu Wang, Chanchan Yu, Yahong Chai, Jin Zhang, Wenchao Li, Jing Liu, Hongxia Sun, Li Yao

**Affiliations:** ^1^ State Key Laboratory for Structural Chemistry of Unstable and Stable Species Institute of Chemistry CAS Research/Education Center for Excellence in Molecular Sciences Chinese Academy of Science Beijing 100190 P. R. China; ^2^ University of Chinese Academy of Sciences Beijing 100049 P. R. China; ^3^ University of Texas Southwestern Medical Center Dallas TX 75390 USA; ^4^ Department of Thoracic Surgery China‐Japan Friendship Hospital Beijing 100029 P. R. China; ^5^ Senior Department of Pediatrics The Seventh Medical Center of Chinese People's Liberation Army General Hospital Beijing 100007 P. R. China; ^6^ Fudan University Shanghai Cancer Center Shanghai 200032 P. R. China

**Keywords:** cancer therapy, mechanical force, mechano‐assimilation, minority Paclitaxel‐resistant cancer cells, tumor microenvironment

## Abstract

Chemotherapy remains a prevalent strategy in cancer therapy; however, the emergence of drug resistance poses a considerable challenge to its efficacy. Most drug resistance arises from the accumulation of genetic mutations in a minority of resistant cells. The mechanisms underlying the emergence and progression of cancer resistance from these minority‐resistant cells (MRCs) remain poorly understood. This study employs force‐induced remnant magnetization spectroscopy (FIRMS) alongside various biological investigations to reveal the mechanical pathways for MRCs fostering drug resistance and tumor progression. The findings show that minority Paclitaxel‐resistant cancer cells have enhanced mechanical properties. These cells can transmit high‐intensity forces to surrounding sensitive cells (SCs) through the force transducer, Merlin. This force transmission facilitates the assimilation of surrounding SCs, subsequently strengthening the contraction and adhesion of tumor cells. This process is termed “mechano‐assimilation,” which accelerates the development of drug resistance and tumor progression. Interestingly, disturbances and reductions of mechano‐assimilation within tumors can restore sensitivity to Paclitaxel both in vitro and in vivo. This study provides preliminary evidence highlighting the contribution of MRCs to the development of drug resistance and malignancy, mediated through mechanical interactions. It also establishes a foundation for future research focused on integrating mechanical factors into innovative cancer therapies.

## Graphical abstract

This study provides new perspectives into the interactions between mechanical forces and cellular dynamics within the tumor microenvironment, particularly elucidating how minority Paclitaxel‐resistant cancer cells (MRCs) promote tumor progression and treatment resistance through mechanical forces and also providing effective treatment strategies for restoring tumor sensitivity to Paclitaxel.

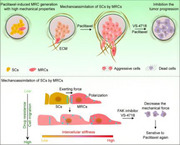

## 1 Introduction

Paclitaxel, also known by its trade name Taxol, is widely used to treat advanced and refractory cancers.^[^ ^1^, ^2^, ^3^ ^]^ However, long‐term cancer treatment with Paclitaxel will generate drug‐resistant cells, which may result in cancer being incurable.^[^ ^4^, ^5^ ^]^ This Paclitaxel resistance is acquired by alterations of tubulin,^[^ ^6^, ^7^, ^8^ ^]^ increase of microtubule dynamics,^[^ ^9^ ^]^ or/and changes of drug‐binding sites,^[^ ^8^ ^]^ all of which are largely mediated by alteration of the biochemical factors.^[^ ^10^ ^]^ However, recent reports have shown that in addition to genetic and biochemical factors, physical interactions and mechanical forces also influence tumor progression and treatment efficacy.^[^ ^11^, ^12^, ^13^, ^14^ ^]^ These mechanical forces involve the stiffness of the extracellular matrix (ECM), shear stress, and tension force, all of which affect tumor mechanics.^[^ ^15^, ^16^, ^17^, ^18^, ^19^, ^20^ ^]^ Therefore, tumors detected through tissue stiffness usually correlate significantly with poor prognosis.

The behavior of cancer cells is mediated by the tension within the intracellular cytoskeleton and ECM.^[^ ^12^ ^]^ For instance, the regulation of ECM stiffness through modifications in collagen crosslinking can affect tumor tissue fibrosis and enhance tumor invasion and metastasis.^[^ ^21^, ^22^ ^]^ The best‐understood model of directed cell migration by ECM stiffness is durotaxis.^[^ ^23^ ^]^ However, the tumor cells inside the tissue are subjected to isometric forces not only from the ECM but also from their neighboring cells.^[^ ^24^, ^25^, ^26^ ^]^ In addition, some external stimuli can also alter the mechanics of tumor cells.^[^ ^27^ ^]^ For example, long‐term therapeutic interventions can result in the generation of Paclitaxel‐resistant cells, accompanied by the alteration of their adhesion ability.^[^ ^10^ ^]^ The rapid emergence of clinical resistance to Paclitaxel is associated with aggressive and incurable forms of cancer. This resistance may be driven by a minority of resistant cancer cells that have altered biomechanical properties.^[^ ^28^, ^29^ ^]^ While mechanical interactions between tumors and their microenvironment are well‐documented, how minority drug‐resistant cells propagate resistance through physical forces remains unexplored. Furthermore, effectively treating drug‐resistant cancers continues to pose a significant challenge.

Force‐induced remnant magnetization spectroscopy (FIRMS) is an innovative technology that integrates scanning magnetic imaging with external perturbation forces such as centrifugation, ultrasound, and microfluidics.^[^ ^30^ ^]^ This technology offers exceptional resolution, enabling the detection of individual DNA base pairs without the need for cumbersome physical separation methods. Recent enhancements to FIRMS have enabled investigations into specific adsorption on cell surfaces, the interaction forces between antibody‐antigen complexes, and the molecular mechanisms governing motor proteins involved in protein synthesis.^[^ ^31^, ^32^, ^33^, ^34^ ^]^ FIRMS stands out due to its immunity to ambient light interference, which ensures the reliability and stability of the magnetic signals obtained. It allows for the simultaneous measurement of numerous single‐molecule events. This significantly reduces the variability of individual measurements and improves experimental reproducibility.

**Scheme 1:**
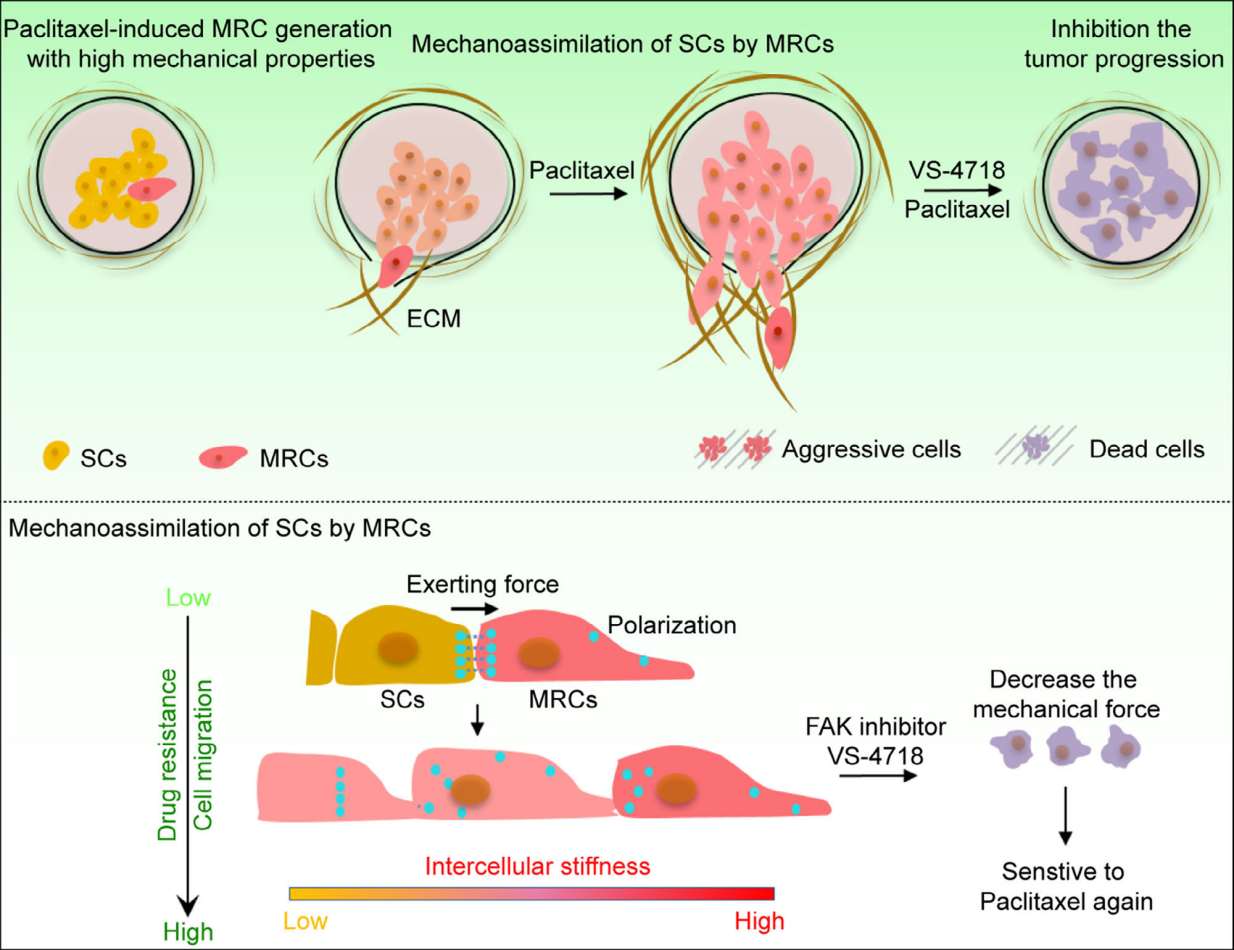
The scheme shows that MRCs increase drug resistance and metastasis of SCs through mechano‐assimilation, and the use of VS‐4718⊂L reduces cellular contraction, thereby restoring the sensitivity of SMRCs to Paclitaxel.

In this study, we employed FIRMS in conjunction with diverse biological investigations to elucidate the role of minority Paclitaxel‐resistant cancer cells (MRCs) as mechanical drivers facilitating tumor drug resistance and progression. Our findings show that MRCs exert significant mechanical forces on nearby sensitive cells (SCs) through the force transducer Merlin. This triggers a cascade of intracellular events, such as actin polymerization and the activation of myosin II within the SCs. Myosin II subsequently acts as another mechanical driver to assimilate the contraction of their surrounding cells, enhancing the contraction and adhesion strength of all the mixed cells (SMRCs). We term this process as mechano‐assimilation. The assimilated SMRCs exhibit distinct features of Paclitaxel resistance and increased migration, invasion, and ECM reconstruction capabilities. These traits collectively enhance the aggressive nature of cancer. Notably, the application of the focal adhesion kinase (FAK) inhibitor VS‐4718 targets mechano‐assimilation, effectively disrupting FAK‐mediated SMRC contraction, thereby reducing tumor cell migration and invasion. This intervention not only prevents SMRCs from rebuilding their ECM but also restores their sensitivity to Paclitaxel (**Scheme** **1**). These findings underscore the critical role of mechanical forces exerted by minority cancer cells in promoting both Paclitaxel resistance and tumor progression.

## 2 Results and Discussion

### 2.1 Paclitaxel‐Resistant Cells Display Higher Mechanical Properties than SCs

**Figure 1:**
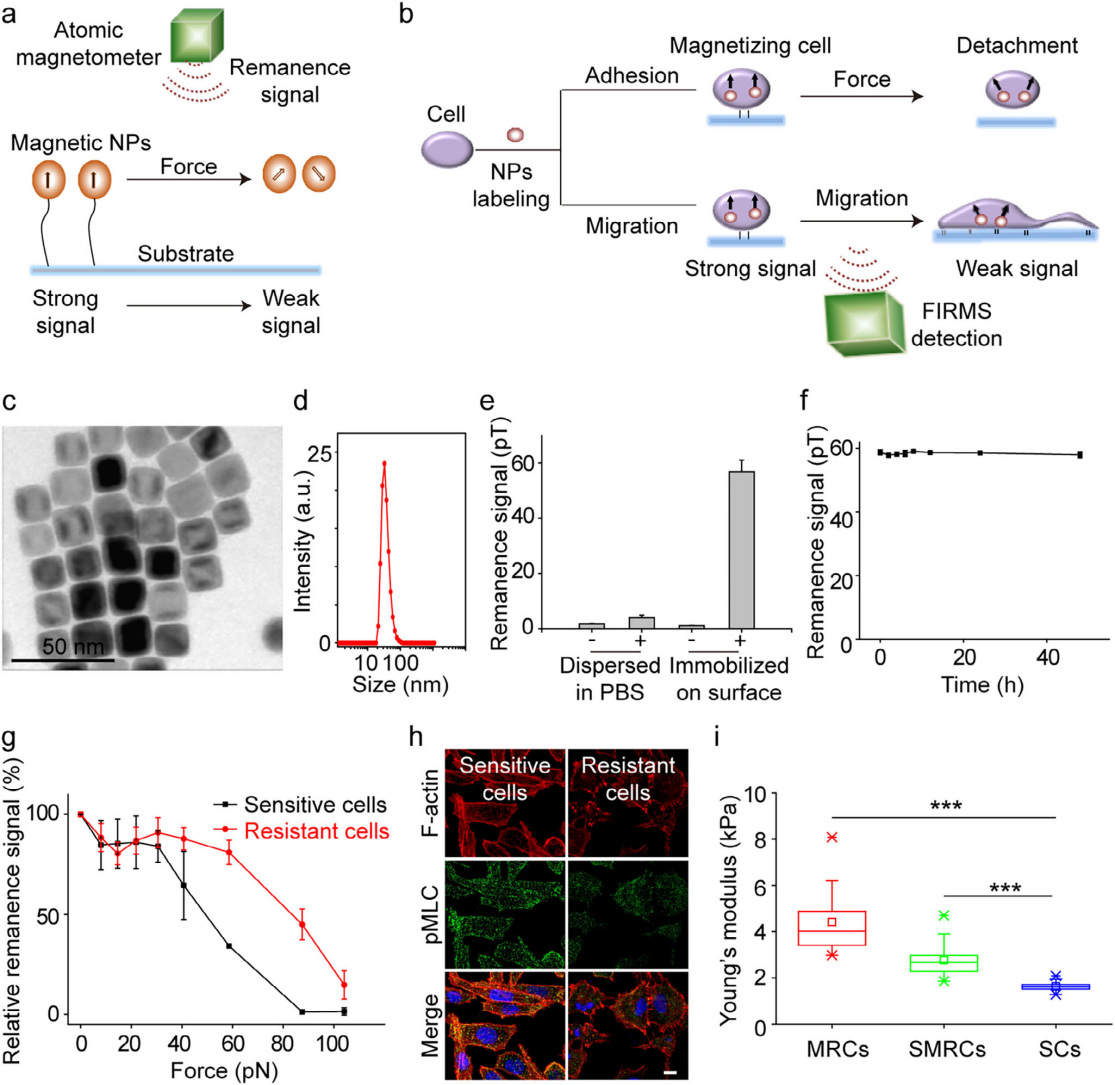
FIRMS and magnetic NPs for measuring mechanical forces. a) FIRMS working profiles. b) Schematic of measuring cell adhesion and migration through FIRMS. c) and d) Optimized nanoprobes (20 nm) characterized by using transmission electron microscopy (TEM) and dynamic light scattering (DLS). e) Magnetic signals of the magnetized NPs (10 µg). “+” and “−” represented NPs with and without magnetization, respectively. f) Time‐dependent signal stability of nanoprobes (10 µg) immobilized on the slice surface. Remanence signal was monitored by FIRMS from 1 h to 48 h after magnetization. g) Curves of force‐dependent relative signal decrease of resistant and sensitive cells. Data are mean ± SD (*n* = 3). h) The actomyosin contractility in resistant and sensitive cells, with F‐actin (red), Serine 19‐phosphorylated myosin II regulatory light chain (pMLC) (green), and nuclei (Blue) staining. Scale bar: 10 µm. i) Young's modulus of SCs, SMRCs, and MRCs investigated with an atomic force microscope (AFM). Data are mean ± SD, n ≥ 300 cells. ***p < 0.001.

Paclitaxel resistance dramatically alters the adhesion capabilities of cancer cells.^[^ ^10^ ^]^ We wondered whether the mechanical properties are different between Paclitaxel‐resistant cells and sensitive cells. To this end, the adhesion strength, intracellular contraction, and Young's modulus between Paclitaxel‐resistant human lung cancer cells (A549‐P) and their sensitive counterparts (A549) were compared. The adhesion strength was measured by using FIRMS in conjunction with magnetic nanoprobes (NPs) (**Figure** **1**a,b).

To conduct this experiment, nanoprobes ranging from 4 to 80 nm were evaluated to see which ones were suitable for FIRMS measurements (Figure S1, Supporting Information). Ultimately, 20‐nm nanoprobes with uniform size (20 ± 2 nm) and high dispersity (PDI = 0.16) were selected for further use (Figure 1c,d; Figure S2, Supporting Information). These nanoprobes exhibited stable remanence signals (Figure 1e,f), excellent biocompatibility, and a good linear correlation between nanoprobe concentration and remanence signals (Figure S3, Supporting Information). Utilizing these nanoprobes, the cellular adhesion forces have been directly and sensitively measured through FIRMS (Figure S4, Supporting Information). The results indicated that the adhesion force of Paclitaxel‐resistant cells was higher than that of sensitive cells (Figure 1g), while the adhesion strength of the mixed population of resistant and sensitive cells fell between these two values (Figure S5, Supporting Information). The intracellular contractility generated by networks of filament actin (F‐actin) and myosin II was also higher in Paclitaxel‐resistant cells than in sensitive cells. Compared to sensitive cells, resistant cells showed greater contraction, marked by more stress fibers and higher myosin II activity (indicated by Serine 19‐phosphorylated myosin II regulatory light chain, pMLC). In addition, resistant cells also displayed extensive integration of myosin minifilaments within F‐actin networks (Figure 1h). The elastic modulus was measured at 4.42 ± 1.36 kPa for the minority Paclitaxel‐resistant cells (MRCs), in contrast to 1.63 ± 0.19 kPa for the sensitive cells (SCs) (Figure 1i). Taken together, these results show that Paclitaxel‐resistant tumor cells display stronger mechanical properties, including greater adhesion strength, increased contraction, and higher intracellular stiffness, in comparison to their sensitive counterparts.

### 2.2 MRCs Enhance the Adhesion Strength and Contraction of Co‐Cultured SCs

**Figure 2:**
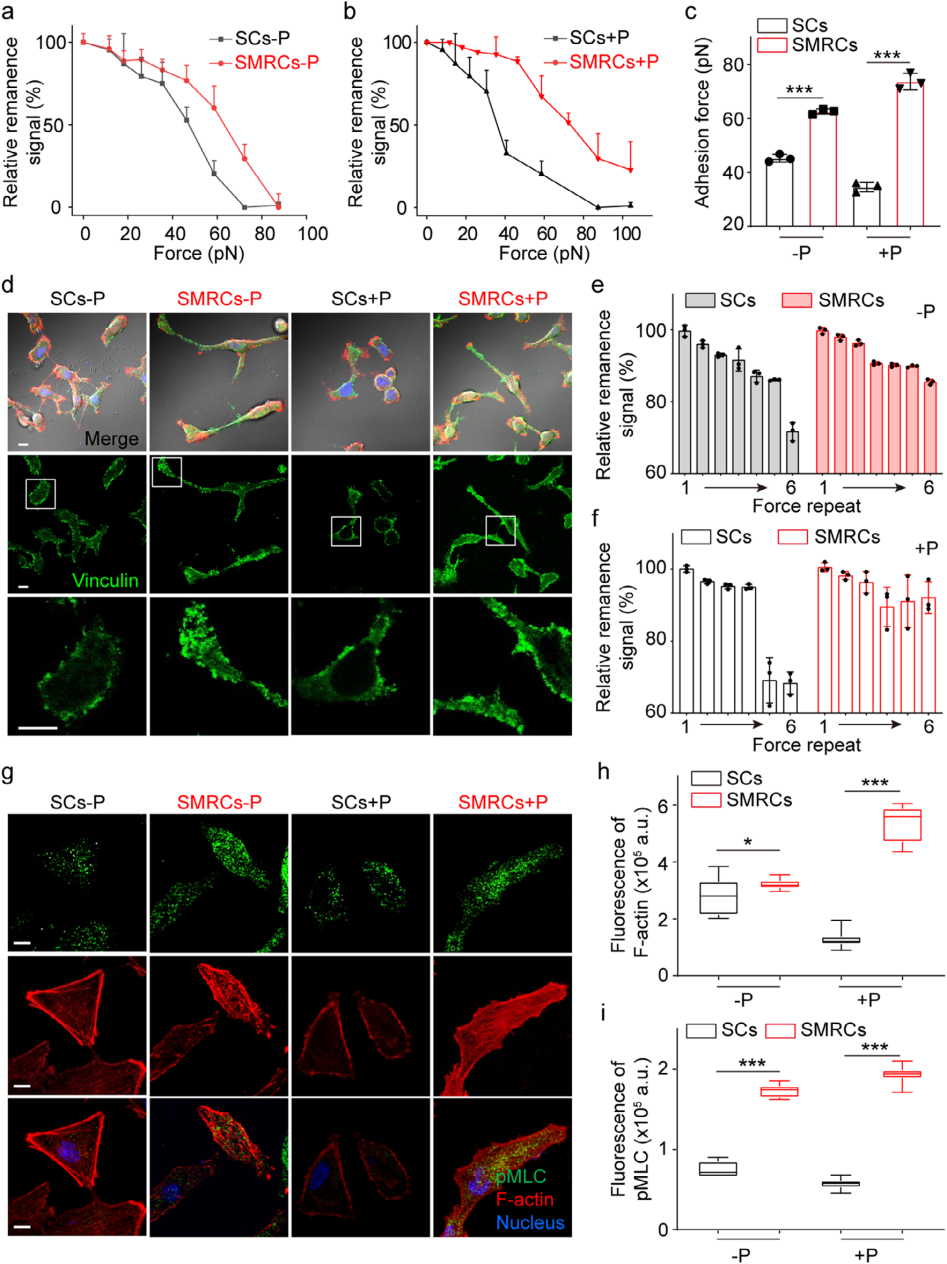
MRCs enhance the mechanical force of SCs. a) and b) Curves of force‐dependent relative signal decrease of SMRCs and SCs treated without (‐) (a) or with (+) (b) Paclitaxel for 36 h, with sample sizes of *n* = 3. c) Quantification of cell adhesion forces from the adhesion force curves shown in (a) and (b). Data are mean ± SD (*n* = 3). d) Representative distributions of FAs (marked by Vinculin) in both SCs and SMRCs under different treatments. F‐actin is shown in red, nuclei in blue, Vinculin in green, and bright field imaging in gray. Scale bar: 10 µm. e) and f) Adhesion stability measured via FIRMS during the application of a constant force of 25 pN across six iterations on cells without (e) and with (f) Paclitaxel treatments. Data are mean ± SD (*n* = 3). g) Confocal images of F‐actin (Red) and pMLC (Green) in cells exposed to various treatments. Scale bar: 10 µm. h) and i) Quantification of F‐actin intensity (h) and myosin II activity (i) in cells with different treatments, based on measurements from over 200 cells. *p < 0.05, ***p < 0.001, determined by Student's *t*‐test.

Given that the rapid emergence of clinical drug resistance may initially arise from MRCs, we sought to determine whether these cells could influence their mechanical properties within the tumor microenvironment. A co‐culture system was constructed according to previous reports to mimic the therapy‐induced heterogeneous tumor tissues containing a minority of resistant cells.^[^ ^28^ ^]^ This system consisted of 99.5% SCs and 0.5% MRCs, resulting in a mixture known as SMRCs. Interestingly, it was found that SMRCs exerted higher adhesion force (≈62 pN) than SCs (≈50 pN) (**Figure** **2**a–c). However, when the contractility of MRCs was inhibited by blebbistatin (a selective non‐muscle myosin II inhibitor) before co‐culture, the adhesion force of SMRCs decreased to ≈50 pN (Figure S6, Supporting Information). This value was similar to that of SCs. This suggests that the significant enhancement in the adhesion force of SMRCs is induced by the contractility of MRCs. Furthermore, when cells were pre‐treated with an integrin‐blocking peptide (PLGVRGRGD)^[^ ^35^ ^]^ or cultured on the bare slice without fibronectin modification, the adhesion force of SMRCs was distinctively reduced to ≈25 pN (Figure S7, Supporting Information). This reduction corresponds to non‐specific adsorption and indicates that the measured adhesion force is dependent on integrin‐fibronectin interactions.

The adhesion strength is correlated with the size and adhesion stability of focal adhesions (FAs), particularly in the context of integrin‐dependent adhesion.^[^ ^36^, ^37^, ^38^ ^]^ We examined vinculin, a crucial protein that links FA to the actin network, and found an increase in FA area within SMRCs, specifically in cells subjected to Paclitaxel treatment (Figure 2d; Figure S8, Supporting Information). The adhesion stabilities were also evaluated by repeatedly exerting a constant force (≈25 pN) on nanoprobe‐labeled cells. With increasing the times of force application, the remanence signal of SCs decreased more rapidly from five to six force applications than that of SMRCs. After six applications of force, the number of SMRCs separated from substrates was obviously less than that of SCs (Figure 2e,f; Figure S9, Supporting Information), suggesting that SMRCs have higher adhesion abilities than SCs. Furthermore, RNA sequencing and Reverse Transcription‐Quantitative Polymerase Chain Reaction (RT‐qPCR) were used to comprehensively characterize the genetic variations of SMRCs relative to SCs (Figures S10 and S11, Supporting Information). The results of both techniques suggest that the enhanced adhesion strength of SMRCs may be associated with alterations in the activity of certain genes.

The maturation and stability of FAs require the reinforcement of the integrin‐actomyosin binding, which in turn facilitates cell contraction.^[^ ^39^ ^]^ Thus, we conducted a detailed investigation into the levels and spatial organization of actomyosin networks. The results revealed that SMRCs contain more F‐actin and myosin minifilaments networks compared to SCs, along with higher myosin II activity and greater integration of myosin minifilaments into F‐actin networks (Figure 2g–i). These findings indicate that SMRCs exhibit higher contractility than SCs. The enhanced contraction force in SMRCs relative to SCs was directly measured using a micro‐fabricated elastic micropillar (Figure S12, Supporting Information).^[^ ^40^, ^41^ ^]^ The change in the elasticity of SMRCs was also confirmed by measuring the Young's modulus using an AFM (Figure 1i). The cell counts of SMRCs and SCs were similar, and no changes were observed in subpopulation distributions within SMRCs or in the ECM after culture (Figure S13, Supporting Information). This indicates that the enhanced mechanical properties of SMRCs were not caused by an increase in the number of MRCs within the co‐culture system. From the results of inhibiting MRC contractility before co‐culture and reducing the actomyosin network of SMRCs (Figure S14, Supporting Information), it is concluded that MRCs increase the intracellular contraction of SCs in a co‐culturing system.

### 2.3 MRCs Enhance the Contraction of SMRCs through Mechano‐Assimilation

**Figure 3:**
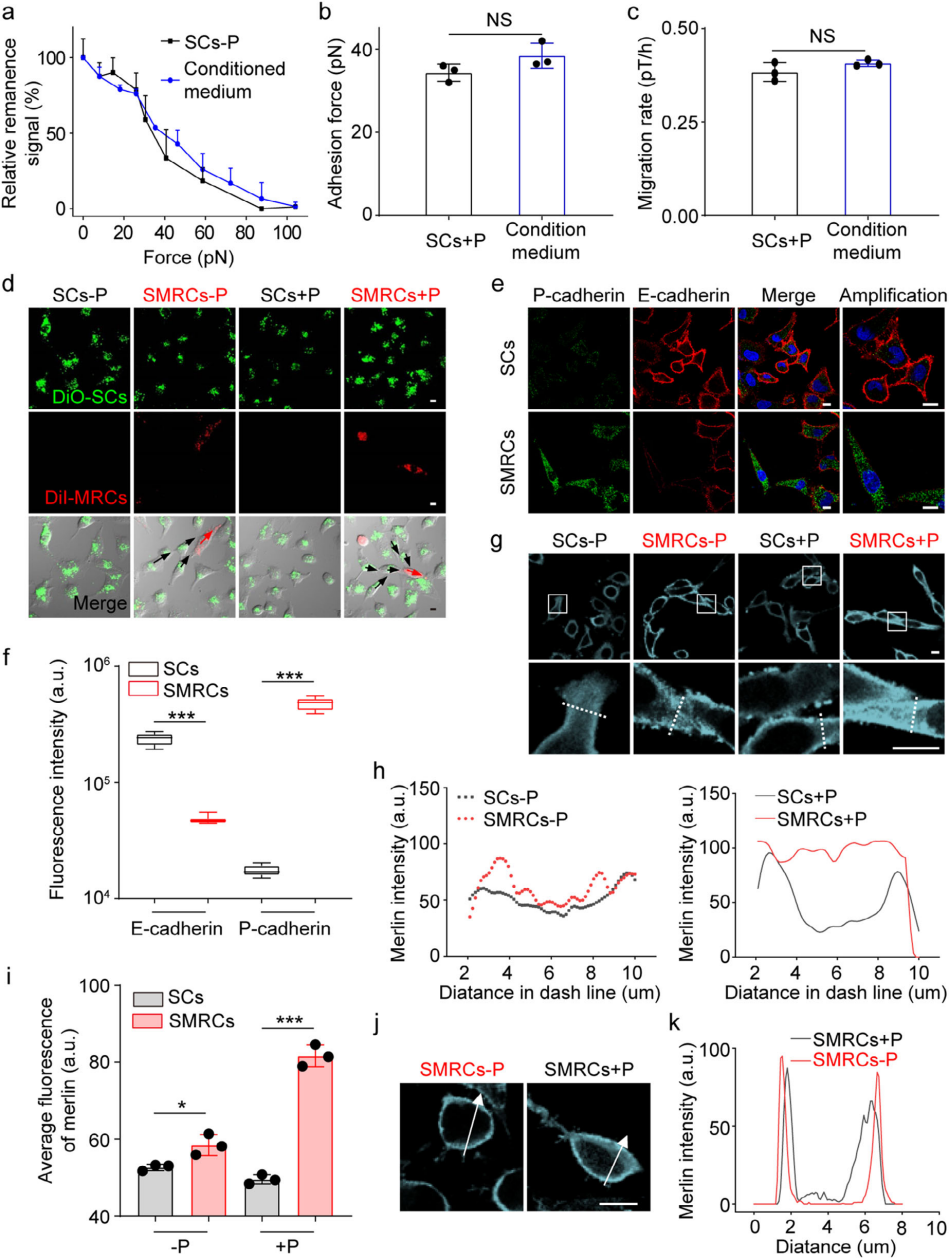
MRCs enhance the contraction of co‐cultured SCs through mechano‐assimilation. a) Curves of force‐dependent relative signal decrease of SCs treated with Paclitaxel and conditioned medium. b) and c) Adhesion force (b) and migration rates (c) of SCs treated with Paclitaxel and conditioned medium measured by FIRMS. Data are mean ± SD (*n* = 3). d) Representative images of cells. Before co‐incubation, MRCs and SCs were stained for 5 min with DiI and DiO, respectively. Scale bar: 10 µm. The dashed arrow indicates the orientation of polarized SCs (black) and MRCs (red). e) and f) Expression levels of E‐ and P‐cadherin in SCs and SMRCs, quantified by fluorescent intensity using ImageJ software. Scale bar: 10 µm. g) Immunofluorescence images of Merlin in cells under different treatments (top) and local magnification in the dashed square (bottom). Scale bar: 10 µm. h) Representative Merlin distribution on the white dashed lines in (g, bottom). i) Quantification of Merlin in cells, represented by average fluorescence intensity. j) and k) Immunofluorescence images of Merlin (j) and its distribution in SMRCs (k), where MRC contractility was inhibited by blebbistatin (+B, 50µM) before mixing with SCs. Scale bar: 10 µm. *p < 0.05, ***p < 0.001, Student's t‐test. *n* = 3 independent experiments.

To gain insight into how MRCs enhanced the contraction and adhesion strength of SMRCs, the effects of soluble factors in the conditioned medium from pre‐cultured Paclitaxel‐resistant cells were evaluated. The result showed that the soluble factors or secretions generated by MRCs had no significant effect on enhancing the adhesion strength and migration of SCs (**Figure** **3**a–c). Therefore, we hypothesize that MRCs may directly transmit physical forces to SCs. The severely polarized MRCs directly interacted with SCs and guided their polarization (Figure 3d). This guidance disappeared after the contractile force of MRCs was inhibited (Figure S15, Supporting Information). These physical interactions between cells are regulated by adjacent adhesion proteins, e.g., E‐ and P‐cadherin.^[^ ^42^, ^43^ ^]^ It has been confirmed that the E‐cadherin expression in SMRCs was significantly decreased compared to that in SCs (Figure 3e,f), which is thought to enable metastasis.^[^ ^44^ ^]^ Contrarily, the expression of P‐cadherin was distinctly increased in the cytoplasm of SMRCs (Figure 3e,f), which stimulated cell‐cell adhesion and aggregation,^[^ ^45^, ^46^ ^]^ leading to an enhancement of the intercellular interaction force among SMRCs (Figure S16, Supporting Information).

The intercellular pulling forces exerted by cells will trigger Merlin to transmit force from high‐mechanical cells to low‐mechanical cells.^[^ ^47^ ^]^ During this transduction process, Merlin is translocated from the cell membrane to the cytoplasm.^[^ ^48^ ^]^ In SMRCs, a fraction of Merlin was observed to be translocated to the cytoplasm, whereas in SCs, Merlin predominantly localized in the boundaries (Figure 3g–i). There was no obvious alteration of Merlin in SCs and SMRCs with inhibiting the contraction of MRCs, and there was no difference in the distribution of phosphorylated‐Merlin in SMRCs and SCs (Figure 3j,k; Figure S17, Supporting Information), indicating that only Merlin underwent dynamic localization and force transduction. Merlin has been proposed to form a complex with focal adhesion kinase (FAK) to regulate various cellular activities, including communication, adhesion, and motility.^[^ ^49^ ^]^ The loss of Merlin is currently under investigation as a potential biomarker for FAK inhibitors.^[^ ^49^, ^50^ ^]^ Here, VS‐4718, a known FAK inhibitor, was employed to block Merlin's function,^[^ ^50^ ^]^ leading to the disassembly of polymerization and F‐actin in SMRCs (Figure S18, Supporting Information). This intervention also markedly affected the activity and distribution of myosin II. The low‐activated myosin II was concentrated near the nucleus instead of inserted into the disassembled F‐actin bundles, indicating that VS‐4718 decreased the contractility of SMRCs. The contraction of SMRCs was significantly decreased, and the cellular morphology was altered from spindle to polygon, similar to SCs (Figure S18, Supporting Information). Based on these results, we propose a mechano‐assimilation mechanism where MRCs act as mechanical drivers. They transmit high levels of contraction to the actomyosin cytoskeleton of adjacent SCs through Merlin, enhancing SC contraction and assimilating surrounding cells, which further increases the stiffness of SMRCs.

### 2.4 SMRCs Exhibit High Migration and Invasion

**Figure 4:**
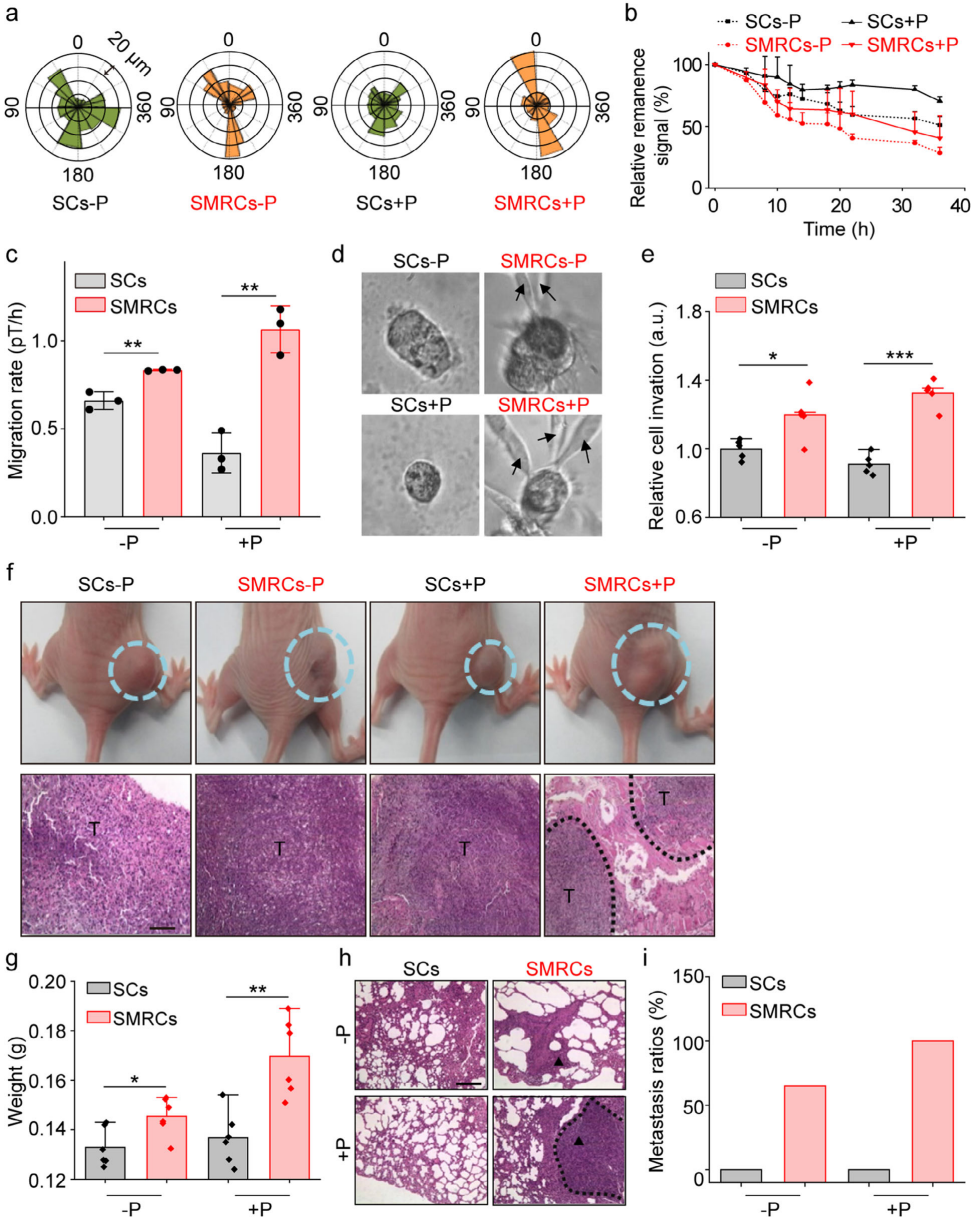
MRCs promote tumor cell invasion and metastasis both in vitro and in vivo. a) A representative angular graph illustrating cell polarization under different treatments. b) and c) Curves of migration‐induced signal decrease recorded by FIRMS (b) and cell migration rates obtained from linear simulation after 36 h treatment (c). Data are mean ± SD (*n* = 3). d) Images of representative tumor‐spheres and individual cells invading from tumor‐spheroids. Arrows: invading cells. e) The relative cell invasion obtained from a transwell invasion assay. Data are mean ± SD (*n* = 5). f) Representative images of tumors (dotted cyan circle) in mice subcutaneously engrafted with cells (SCs and SMRCs) (Top). These mice were treated with 200 µL PBS or Paclitaxel (0.2 mg mL^−1^) for 25 days. Representative hematoxylin and eosin (H&E) staining images of tumor tissue (Bottom). T: tumor. Scale bar: 500 µm. g) Average lung weights from mice subjected to tail vein injection with SCs and SMRCs, respectively. Data are mean ± SD (*n* = 6). h) H&E staining images of lung tissues from mice under different treatments. Black triangles indicate notable metastatic foci. Scale bar: 500 µm. i) Metastasis ratios were calculated as the percentages of mice with metastasis relative to the total number of mice in each group, with 6 mice per group. *p < 0.05, **p < 0.01, ***p < 0.001, assessed by Student's t‐test.

Cell migration is fastest when the adhesion strength between cells and substrates is neither too strong nor too weak.^[^ ^51^ ^]^ It is curious how the migration rate and invasion ability of SMRCs have changed. It was detected that SMRCs formed protrusions with high polarization ratios in one or two directions (‐Paclitaxel, 67 ± 15%; +Paclitaxel, 75 ± 16%), which facilitates their persistent movement in the same direction. This is different from SCs, which displayed multiple protrusions with relatively lower polarization ratios (‐Paclitaxel, 42 ± 12%; +Paclitaxel, 16 ± 2%) (**Figure** **4**a; Figure S19a, Supporting Information). Direct measurements of cell migration rates, conducted using FIRMS to track the reduction in migration‐induced remanence signals, confirmed that the migration speed of SMRCs was ≈3.4‐fold (+Paclitaxel) higher than that of SCs under identical conditions (Figure 4b,c). The remanence signal in glutaraldehyde‐fixed cells remained stable for 36 h (Figure S19b, Supporting Information). This indicates that the decrease in remanence signal was mainly caused by cell migration rather than the Brownian motion of the nanoprobes within cells, which aligns with earlier studies.^[^ ^52^ ^]^ Additionally, cell migration rates were further evaluated through wound healing and transwell migration assays (Figures S19c and S20, Supporting Information). Taken together, these results indicate that MRCs significantly increase the migration rate of co‐cultured SCs.

SMRCs displayed distinct polarization and an asymmetrical distribution of FAs (Figures 4a, and 2d). Additionally, a loss of E‐cadherin was observed (Figure 3e,f), which may accelerate cell motility and promote the detachment of cells from the primary tumor during early invasion and dissemination (Figure S21, Supporting Information). The enhanced invasion of SMRCs, as influenced by MRCs, was further corroborated through direct observations of cell invasiveness in a 3D culture system (Figure 4d,e). Notably, in SMRC‐tumor spheroids, multiple cells were observed invading the spheroids, a phenomenon that was significantly enhanced following treatment with Paclitaxel. Conversely, no invasive cells were detected in SC‐tumor spheroids.

The enhanced cell contraction and adhesion strength, as well as invasion/dissemination, might increase the invasion/metastasis of SMRCs in vivo. To investigate this further, mouse models with subcutaneous tumor xenografts were constructed to examine the invasion capabilities of both SMRCs and SCs in vivo. SMRC‐bearing mice exhibited a markedly more conglomerated tumor morphology compared to SC‐bearing mice (Figure 4f). It is well documented that during the initial stage of metastasis, tumor cells generally invade surrounding tissues and form an invasive tumor ball.^[^ ^53^ ^]^ The histopathological images of tumor tissue revealed that SMRC tumors developed a greater number of invasive lesions (Figure 4f).

To further investigate the role of MRCs in tumor metastasis, a murine metastatic model was employed. Mice subjected to tail vein injection with SMRCs exhibited increased lung weight compared to those injected with SCs (Figure 4g). Notably, the lungs of mice injected with SMRCs displayed more aggressive metastatic foci, exhibiting higher metastasis ratios (65% for SMRCs‐Paclitaxel and 100% for SMRCs+Paclitaxel) compared to SC mice, which showed no metastasis foci (Figure 4h,i; Figure S22a, Supporting Information). Furthermore, SMRCs exposed to Paclitaxel also resulted in the formation of metastatic foci in the kidneys (Figure S22a, Supporting Information), an occurrence indicative of aggressive cancer and advanced disease stages, despite the relative rarity of renal metastasis.^[^ ^54^ ^]^ Consistent with expectations, mice injected with SMRCs, where the contraction of MRCs was inhibited before injection, did not show any significant metastatic foci in their organs (Figure S22b, Supporting Information). Taken together, these results suggest that the metastasis of SMRC tumors was significantly promoted by MRCs.

### 2.5 Mechanisms for Enhanced SMRC Tumor Metastasis In Vivo

**Figure 5:**
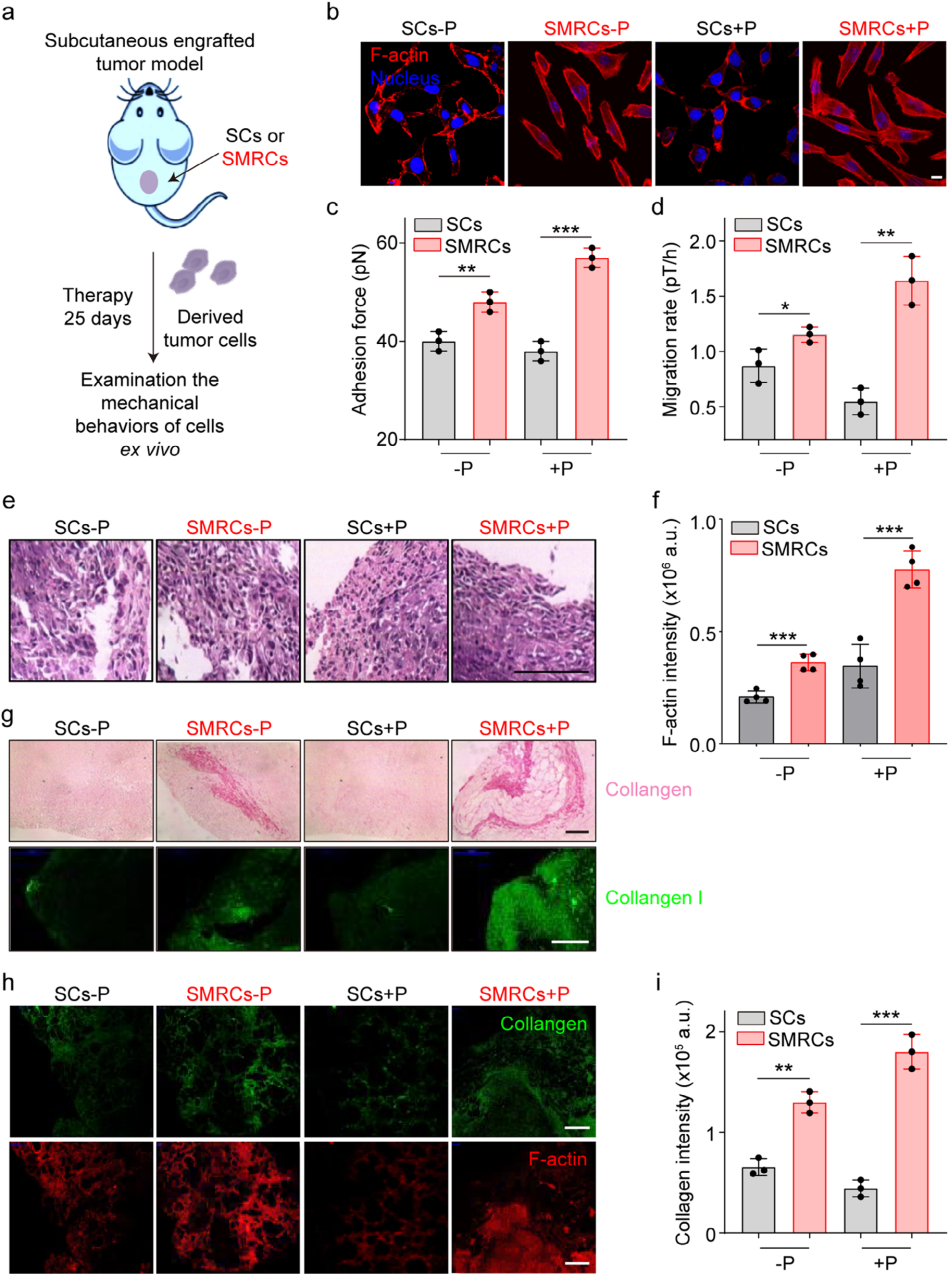
MRCs promote invasion and metastasis by enhancing tumor cell contraction and rebuilding their ECM. a) Schematic representation of the experimental design, utilizing tumor cells derived from subcutaneous tumor xenografts after 25 days of treatment. b) Representative microscopy images show significant polarization of tumor cells isolated from mice with subcutaneous tumor xenografts. Cells were stained for F‐actin (Red) and nuclei (Blue). Scale bar: 10 µm. c) and d) Adhesion forces (c) and migration rates (d) of tumor cells were assessed ex vivo using FIRMS. Data are mean ± SD (*n* = 3). e) Representative H&E staining of cells in tumor margin. Scale bar: 500 µm. f) Quantitative analysis of relative fluorescence intensity of F‐actin within tumor tissue. Data are mean ± SD (*n* = 4). g) Representative images of Sirius Red staining for fibrotic collagen (top) and immunofluorescence staining for collagen I in tumor tissue from mice with different treatments (25 days) (bottom). Scale bar: 500 µm. h) Immunofluorescence staining images of collagen (top) and Phalloidin staining images of F‐actin (down) in lung tissues from mice under different treatments. Scale bars: 500 µm. i) Quantification of collagen in lung tissue from mice subjected to various treatment conditions. Data are mean ± SD (*n* = 3). *p < 0.05, **p < 0.01, ***p < 0.001.

To investigate the mechanisms underlying the enhanced SMRC invasion and metastasis in vivo, the polarization and migration rates were measured in the tumor cells derived from SC‐ and SRMC‐tumor‐bearing mice (Figure**5**a). Compared to SC tumor cells, SMRC tumor cells showed distinct polarization under the enhanced polymerization and the drive of actin stress fibers (Figure 5b), which was more pronounced than that observed in vitro. The adhesion force and migration capacity of cells derived from SMRC‐tumor‐bearing mice were markedly elevated (Figure 5c,d). Using microfluidic systems to mimic vascular systems, it was found that SMRCs were able to withstand greater shear forces and exhibited a tendency to adhere more readily to the walls of fibronectin‐coated microchannels (Figure S23, Supporting Information). Importantly, adhered SMRCs easily formed clusters within these microfluidic channels. The existence of such clusters in blood vessels can promote metastasis.^[^ ^55^ ^]^ This clustering is likely attributable to enhanced intercellular interaction forces among SMRCs, which are regulated by increased levels of P‐cadherin (Figure 3e,f).

At the tissue level, actin polymerization and polarization in the tumor margin cells of SMRC‐bearing mice were more pronounced than in SC‐bearing mice, which increased the probability of tumor cells moving to nearby normal tissues (Figure 5e,f; Figure S24, Supporting Information). It is noteworthy that SMRC‐bearing mice have a higher extent of collagen deposition in both tumors and metastatic foci (Figure 5g–i), indicating that SMRCs with enhanced contraction and adhesion strength can rebuild their ECM. This reorganization is likely to facilitate a positive feedback loop that promotes the invasion and metastasis of tumor cells in vivo. Consequently, the enhanced contraction and adhesion strength of SMRCs promoted tumor metastasis in vivo.

### 2.6 Acquisition of Paclitaxel Resistance by SMRC Tumors

Of particular note, the presence of MRCs markedly increased the biomechanical properties of co‐cultured SCs, rendering Paclitaxel no longer effective for SMRC tumors both in vitro and in vivo. The half‐maximum inhibition concentration (IC50) of Paclitaxel for SCs was approximately 7 nm, whereas the IC50 for SMRCs increased to ≈35 nm, representing a fivefold increase (Figure S25a, Supporting Information). This elevation in IC50 indicates that SMRCs have developed resistance to the drug. Additionally, the microtubules targeted by Paclitaxel in SMRCs dynamically switched from a growth phase to a shrinking phase within 30 min (Figure S25b, Supporting Information), implying that SMRCs exhibit higher intracellular contractility compared to SCs.^[^ ^56^ ^]^ Thus, we propose that the acquisition of Paclitaxel resistance in SMRCs is primarily attributed to an enhancement in intracellular contractility mediated by the mechano‐assimilation of MRCs.

### 2.7 Restoring SMRC Sensitivity to Paclitaxel by Interfering with Contraction and Adhesion

**Figure 6:**
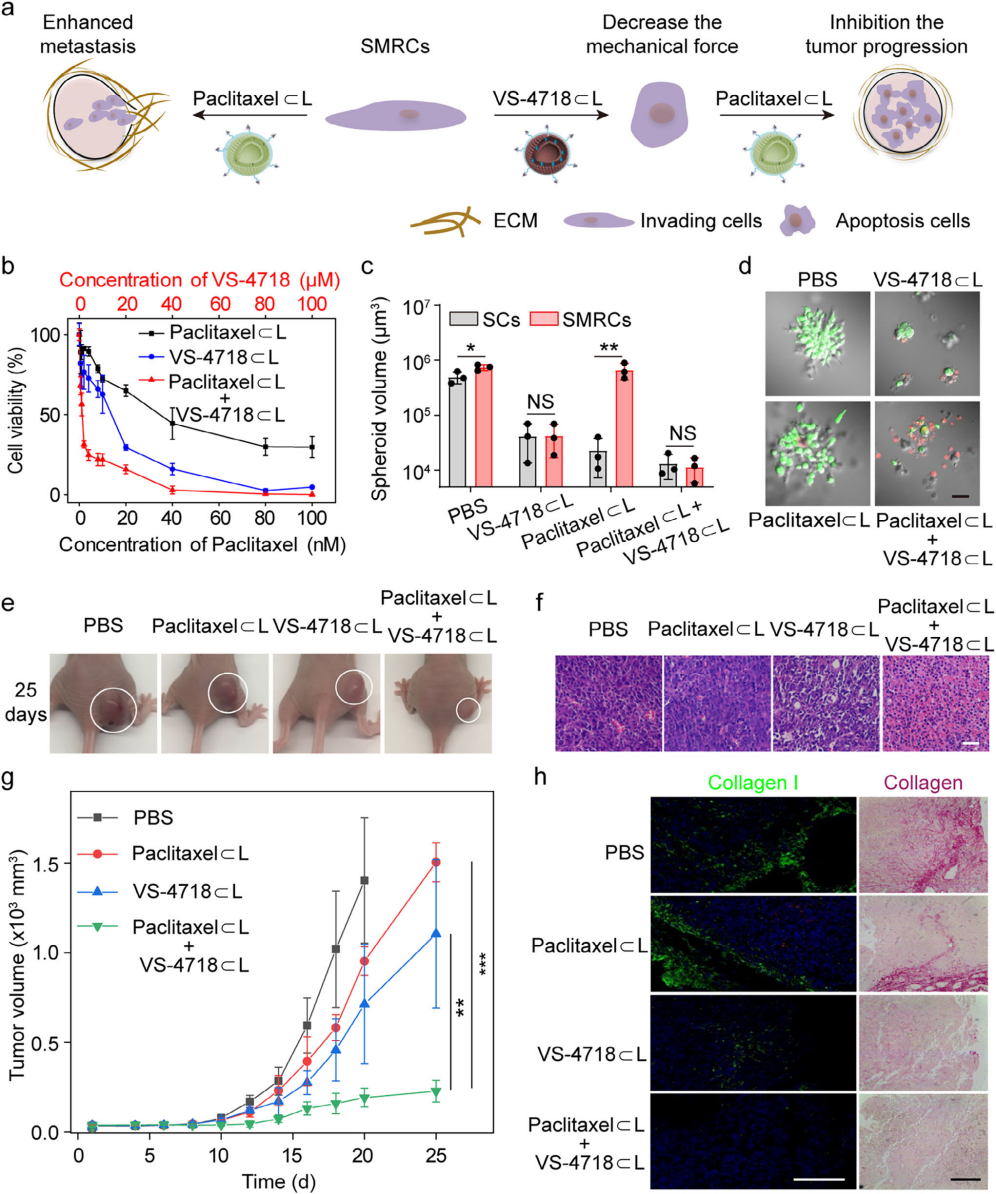
Restoration of Paclitaxel sensitivity in SMRCs through mechanical force reduction. a) The schematic illustrates that further therapy is invalid for SMRC tumors, whereas decreasing the mechanical force of SMRCs using VS‐4718⊂L restores their sensitivity to Paclitaxel. b) Relative cell viability of SMRC cells after treatment with Paclitaxel⊂L, VS‐4718⊂L, together with Paclitaxel⊂L and VS‐4718⊂L, compared to the PBS control. c) Volumes of 3D spheroids of SCs and SMRCs under varying treatment conditions. Data are mean ± SD (*n* = 3). d) Evaluation of growth and activity of SMRC spheroids via live/dead cell staining, Scale bar: 200 µm. e) Representative images of tumor sizes from four groups of mice after 25 days of treatment. f) Representative images of H&E staining in tumor margins from mice subjected to different treatments. Scale bar: 500 µm. g) The volumes of SMRC‐xenografted tumors over time under various treatments, wherein the mice received intravenous injections of agents on days 11, 13, 15, 17, and 19. Data are mean ± SD (*n* = 5). h) Immunofluorescence staining for collagen I (left) and Sirius Red staining for fibrotic collagen (right) in tumor tissues from mice receiving different treatments. Scale bar: 500 µm. *p < 0.05, **p < 0.01, ***p < 0.001.

Given that the application of FAK inhibitors to disturb mechano‐assimilation can decrease the contraction and adhesion strength of SMRCs, we wondered whether such mechanical force reduction would restore the sensitivity of SMRC tumors to Paclitaxel (**Figure** **6**a). Recent advancements in nanomedicine, including stimuli‐responsive drug carriers,^[^ ^57^ ^]^ targeted delivery systems,^[^ ^58^ ^]^ and mechano‐adaptive nanomaterials,^[^ ^59^ ^]^ underscore the utility of liposomal encapsulation in enhancing therapeutic efficacy while minimizing off‐target effects. To explore this hypothesis, the IC_50_ of Paclitaxel in the presence of VS‐4718 encapsulated in liposomes (VS‐4718⊂L, ≈ 75 nm, PDI = 0.28, Figure S26, Supporting Information) was determined. The IC_50_ of Paclitaxel for SMRCs was found to decrease from ≈38 to ≈ 2 nm, signifying a 17‐fold reduction in sensitivity compared to SMRCs treated with Paclitaxel alone (Figure 6b; Figure S27a, Supporting Information). This result indicates a renewed sensitivity of SMRCs to Paclitaxel. Furthermore, in 3D tumor spheroids, the invasion and spheroid growth of SMRCs were significantly inhibited by Paclitaxel⊂L following modulation of their contraction and adhesion via VS‐4718⊂L treatment (Figure 6c,d; Figure S27b, Supporting Information). In contrast, SMRC spheroids treated only with PBS or Paclitaxel displayed larger spheroid volumes and increased cell invasion. The observed suppression of tumor spheroid growth was primarily attributable to an increase in cell death and a reduction in cell migration (Figure 6d; Figures S27 and S28, Supporting Information).

To further validate the restored sensitivity of SMRC tumors to Paclitaxel following the disruption of mechano‐assimilation and the subsequent reduction in contraction in vivo, we examined the therapeutic efficacy of Paclitaxel using the SMRC‐xenograft model. After the formation of tumors, the tumor‐bearing mice received intravenous injections of VS‐4718⊂L and Paclitaxel⊂L. Compared to the control groups (PBS, VS‐4718⊂L, or Paclitaxel⊂L), the combination treatment of VS‐4718⊂L and Paclitaxel⊂L notably suppressed tumor growth (Figure 6e,f). There were no notable changes in the body weights of the mice across the various treatment groups, indicating that the acute toxicity associated with the nanocarriers was minimal (Figure S29, Supporting Information). These results suggest that the disturbance of contraction and adhesion in SMRCs re‐sensitized the tumors to Paclitaxel in vivo. Interestingly, the tissue of SMRC tumors became looser and exhibited reduced collagen I levels after VS‐4718⊂L treatment (Figure 6g,h), indicating that SMRCs, with decreased contraction and adhesion strength, failed to rebuild the ECM. Collectively, these results support the hypothesis that decreasing mechanical force renders the SMRC tumors sensitive to Paclitaxel again, thereby improving the therapeutic efficacy against this resistant cancer both in vitro and in vivo.

## 3 Discussion

Long‐term Paclitaxel therapy is known to induce drug resistance and promote tumor metastasis, yet the molecular and cellular mechanisms underlying these processes remain poorly understood. In this study, we employed high‐throughput FIRMS technology to investigate the mechanical behaviors of tumor cells and identified an unexpected mechano‐assimilation mechanism. This mechanism illustrates how MRCs, acting as mechanical cues, influence drug resistance and metastasis. In tumor tissues, there are many heterogeneous cell types resulting from an accumulation of genetic mutations during tumor progression.^[^ ^60^ ^]^ The mechanical heterogeneity inherent in these tissues has the potential to activate growth factor signaling pathways, thereby promoting tumor malignancy through the modulation of tissue stiffness and fibrosis.^[^ ^61^ ^]^ Such alterations in the tumor microenvironment can influence the mechanical properties and migratory behavior of tumor cells. However, only a minority of cells typically display altered phenotypes. Here, our findings demonstrate that once minority cells with enhanced mechanical properties are generated within tumor tissue, the mechanical behaviors of tumor cells are rapidly assimilated by MRCs. Since the stiffness of MRCs was higher than that of surrounding SCs, cells could assimilate their surrounding cells along with intercellular‐stiffness gradients, we termed this process mechano‐assimilation. During this process, MRCs functioned as leader cells, exerting and transmitting stronger mechanical forces to adjacent SCs through the Merlin protein. This led to increased actin stress fibers and activation of myosin II in SCs. These changes may subsequently transform SCs into new leader cells. While our findings establish Merlin as a critical force transducer in mechano‐assimilation, further research is necessary to elucidate the detailed molecular pathways through which mechanical forces are converted into biochemical signals. In particular, it is essential to investigate how Merlin interacts with downstream effectors, such as Hippo‐YAP/TAZ and integrin‐FAK signaling pathways, to enhance cytoskeletal remodeling and contribute to drug resistance.

The generation of Paclitaxel resistance is dependent on the drug concentrations. Several potential mechanisms have been proposed to explain resistance at different concentrations of Paclitaxel, including increased microtubule dynamics, enhanced microtubule polymerization, alterations in tubulin isotypes, and mutations within tubulin itself. In contrast, our study proposes that SMRCs acquire drug resistance through an intriguing mechano‐assimilation mechanism that operates independently of Paclitaxel concentrations. Once MRCs with enhanced mechanical properties are generated, the drug resistance of SMRCs increases sharply due to the enhanced contractility aroused by actomyosin assemblies, leading to an increase in microtubule dynamics. These findings align with previous reports that indicate a correlation between cell contractility and microtubule dynamics.^[^ ^62^ ^]^ More importantly, further Paclitaxel treatment exacerbates the mechano‐assimilation process prompted by the slight accumulation of microtubules at the leading edges of cells. This microtubule accumulation is regulated by myosin II to balance actomyosin and microtubules, which further increases the polarization capability of cells.^[^ ^63^ ^]^ Therefore, we suggest that Paclitaxel therapy positively contributes to the mechano‐assimilation of MRCs. This finding clarifies why the mechanical behaviors of SMRCs are not impeded but significantly enhanced following Paclitaxel treatment. Collectively, the concept of mechano‐assimilation emerges as a novel mechanism that drives drug resistance, ultimately culminating in clinical relapse during subsequent therapies.

The successful metastasis is dependent, in part, on the mechanical properties of tumor cells, their microenvironment, and their physical interactions. During the detachment and invasion phases of carcinoma cells from primary tumors, the cellular mechanical properties and morphologies show significant changes, leading to the acquisition of a motile phenotype.^[^ ^64^ ^]^ Our data reveal that SMRCs readily adopt a pro‐migratory phenotype induced by MRC‐enhanced mechanical properties and polarization, thereby advancing cell invasion in 3D organoids and in vivo mouse models. This supports the previous prediction that even minimal cell dispersion can substantially increase cell invasion and metastasis.

Invasive tumor cells with enhanced mechanical properties will subsequently encounter an ECM rich in collagen I.^[^ ^65^ ^]^ The mechanical reciprocity between tumor cells and the ECM, alongside their modulation by mechanical forces, plays a pivotal role in tumor metastasis.^[^ ^66^ ^]^ Moreover, cancer cells can also remodel their surrounding ECM which in turn promotes malignancy.^[^ ^67^ ^]^ We observed that MRCs not only assimilated the mechanical properties of SCs but also enhanced actin polymerization and polarization in tumor margin cells. Additionally, ECM stiffness increased due to higher collagen I deposition and the formation of fibers/bundles at tumor margins. These findings imply that the mechanical forces generated by the actomyosin contractility of SMRCs could modulate or remodel the ECM, engendering tumor invasion and metastasis, which is consistent with previous observations that tissue tension and collagen deposition potentiate tumor malignancy.^[^ ^20^ ^]^

The tumor cells are subjected to strong shear forces from blood flow within the circulatory system. Only circulating tumor cells that can overcome fluid shear will adhere to vessel walls, forming metastatic foci.^[^ ^68^ ^]^ Our observations indicate that SMRC tumor cells possess the capability to endure high shear forces and readily adhere to the formerly adhered cells on microfluidic substrates, promoting the formation of cell clusters. A study has shown that the increase of cell clusters within blood vessels greatly contributes to the metastatic spread of cancers.^[^ ^55^ ^]^ In our in vivo metastasis models, SMRC‐injected mice showed sharply increased metastasis ratios. This suggests that MRCs, acting as mechanical potentiating cues, can promote metastasis by enhancing the physical properties of tumor tissues, even during Paclitaxel treatment. Consequently, disturbing mechano‐assimilation will be an efficient approach for re‐sensitizing resistant cancer cells to chemotherapy. As it turned out, the use of agents capable of destroying mechanical forces has indeed improved the efficacy of tumor treatment, signaling a potential avenue for future cancer therapies.

In summary, we show that once minority cells with enhanced contraction are generated within tumor tissues, the mechanical behaviors of tumor cells are rapidly assimilated by MRCs. This process not only promotes drug resistance but also facilitates tumor malignancy and treatment failure. Given that MRCs exhibit greater stiffness than surrounding SCs, we have referred to the phenomenon of these cells assimilating their surroundings along intercellular stiffness gradients as “mechano‐assimilation”. Disturbing the mechano‐assimilation and decreasing the contraction and adhesion strength of SMRCs render these intractable cancer cells sensitive to chemotherapy again. This finding highlights the significant role of mechanical forces in modulating Paclitaxel resistance and tumor progression. Building on these insights, future research should prioritize translational steps, including the dose optimization of VS‐4718/Paclitaxel combinations in immunocompetent models, thorough toxicity assessments, and the validation of biomarkers for patient stratification. Additionally, the use of nanocarrier‐mediated delivery of FAK inhibitors (e.g., VS‐4718⊂L) promises to enhance tumor targeting while minimizing associated side effects.^[^ ^69^ ^]^ Clinical trials that integrate mechanical biomarkers, such as Merlin localization and collagen I deposition, will be instrumental in identifying patients who could benefit from mechano‐targeted therapies. By establishing a link between mechanobiology and clinical oncology, this work opens new avenues to combat recalcitrant tumors resistant to conventional chemotherapy.

## 4 Experimental Section

### Examination of Remanence Signal by Using FIRMS

Nanoparticles with an average diameter of 20 nm were used as optimal nanoprobes. 10 µg nanoprobes were dissolved in PBS and dropped on the FIRMS slice, and finally immobilized on the slice surface by solvent evaporation. The remanence signal was examined through FIRMS after magnetization in the vertical direction for ≈2 min by using a permanent magnet. Control experiments were carried out without solvent evaporation or magnetization. The signal stability of nanoprobes was examined by FIRMS from 1 to 48 h after magnetization. The sensitivity of nanoprobes was investigated in the mass range: 0, 0.5, 1, 2, 4, 8, and 10 µg.

### Slice Modification

The FIRMS slice was designed by utilizing a polydimethylsiloxane (PDMS) slice with a hole (4 mm in diameter, 1 mm in depth). After hydroxylation by plasma cleaner for 25 s, the APTES solution of ethanol was quickly added to the activity surface of PDMS and incubated for 4 h. The slices were subsequently rinsed with solvent and dried under a stream of nitrogen. The silanized surface was activated by incubation in a solution of glutaraldehyde (1% v/v). Then the solution was replaced by fibronectin (FN) solution (20 µg mL^−1^). The slices were kept at 4 °C overnight and subsequently rinsed. Before the FIRMS measurement, the BSA solutions (1% w/v) were added to the slice for the elimination of nonspecific adsorption.

### Detection of Deformability and Adhesion of Magnetically Labeled Cells

The cells were incubated in the FIRMS slice for 17 h. The nanoprobes (20 µg mL^−1^) were added to the cell medium for cell uptaking. The free nanoprobes in the medium were removed and washed with PBS three times. After sample magnetization, 40 µL of trypsin (0.25%) as a mimetic force driver was added to the FIRMS slice. The time‐dependent signal changes induced by cellular deformation and detachment were recorded by FIRMS. Meanwhile, in control experiments, cells were treated under the same conditions in the absence of trypsin, where the cellular signal was kept stable for 2 h. When the nanoprobes‐labeled cells were fixed with glutaraldehyde (4%) for 20 min, the signal was measured.

### Animals

All animal experiments were approved by the Institutional Animal Care and Use Committee of the National Center for Nanoscience and Technology (Protocol No. NCNST21‐2211‐0401). 6 to 8‐week‐old female BALB/c nude mice were obtained from Beijing Vital River Laboratory Animal Technology Co., Ltd.

### Statistical Analysis

Statistical analysis and graphs were performed using GraphPad Prism 8.0 software (GraphPad Software, Inc.). For comparison between the two groups, significance was determined using the two‐tailed unpaired Student's t‐test (*P < 0.05, **P < 0.01, ***P < 0.001). Differences with a minimum *P* < 0.05 were considered statistically significant. All data are presented as mean ± SD.

## Conflict of Interest

The authors declare no conflict of interest.

## Supporting information



Supporting Information

## Data Availability

The data that support the findings of this study are available from the corresponding author upon reasonable request.
