# Supplementary material for: Mechanisms and Therapeutic Strategies for Minority Cell‐Induced Paclitaxel Resistance and Tumor Progression Mediated by Mechanical Forces
Source: Adv Sci (Weinh). 2025 Apr 24;12(22):2417805. doi: 10.1002/advs.202417805 (PMC12165044; doi:10.1002/advs.202417805)
Supplement: Supplementary file 1 — Supporting Information [file ADVS-12-2417805-s001.pdf]

ADVANCED SCIENCE
Open Access

Supporting Information

for *Adv. Sci.*, DOI 10.1002/advs.202417805

Mechanisms and Therapeutic Strategies for Minority Cell-Induced Paclitaxel Resistance and Tumor Progression Mediated by Mechanical Forces

Xueyan Feng, Di Zhang, Guoxun Wang, Liwei Lu, Feng Feng, Xiuyu Wang, Chanchan Yu, Yahong Chai, Jin Zhang, Wenchao Li, Jing Liu, Hongxia Sun and Li Yao**

Supporting Information

Mechanisms and Therapeutic Strategies for Minority Cell-Induced Paclitaxel Resistance and Tumor Progression Mediated by Mechanical Forces

Xueyan Feng, Di Zhang, Guoxun Wang, Liwei Lu, Feng Feng, Xiuyu Wang, Chanchan Yu, Yahong Chai, Jin Zhang, Wenchao Li, Jing Liu, Hongxia Sun and Li Yao**

Experimental Section

Materials

Fetal Bovine Serum (FBS) was purchased from Gibco (Thermo Fisher Scientific Inc.), Penicillin and streptomycin, Phosphate-Buffered Saline (PBS), DMEM medium, and Trypsin obtained from HyClone/Thermo Fisher. (3-aminopropyl) triethoxysilane (APTES), Fibronectin (FN), 50% glutaraldehyde, Gelatinase, Diphenyl Oxide, Iron (III) acetylacetonate ($Fe(acac)_3$), DSPE-PEG-NHS, L-α-Phosphatidylcholine, and cholesterol were purchased from Sigma-Aldrich. Oleylamine and 3, 4-dihydroxyhydrocinnamic acid (DHCA) were all purchased from Sigma-Aldrich (USA). Paclitaxel-resistance A549 (chronically exposed to Paclitaxel (230 nM)) was purchased from Shanghai Gefan Biotechnology. CO, LTD. 4',6-diamidino-2-phenylindole (DAPI) was purchased from Beyotime, China. Cell culture plates were purchased from Coning Company.

Methods

Synthesis of magnetic nanoparticles

4 nm: 4 nm nanoparticles were synthesized according to the previous report.[1]

10 nm: $Fe(acac)_3$ (2 mmol), oleic acid (4 mmol), and 1-octadecene (20 mL) were mixed and magnetically stirred (1600 rpm) under a flow of nitrogen and heated to 300°C (1 h). The black-brown mixture was cooled to room temperature by removing the heat source. Under ambient conditions, ethanol (50 mL) was added to the mixture, and a black material was precipitated and separated via centrifugation (6000 rpm) for 10 min. The black product was dissolved in hexane and centrifuged (6000 rpm) for 10 min. The supernatant was re-precipitated with ethanol 3 times, and finally, the products were dispersed into hexane.

20 nm: $Fe(acac)_3$ (2 mmol), oleic acid (6 mmol), oleylamine (6 mmol), and 1-octadecene (20 mL) were mixed and magnetically stirred (1600 rpm) under a flow of nitrogen and heated to 200°C (30 min), and then heated to 300°C (30 min). The same precipitated method was carried out as that of the treatment of 10 nm NPs.

40 nm: $Fe(acac)_3$ (2 mmol), oleic acid (6 mmol), oleylamine (6 mmol), and 1-octadecene (10 mL were mixed and magnetically stirred (1600 rpm) under a flow of nitrogen and heated to 200°C (30 min), and then heated to 300°C (30 min). A black material was precipitated and separated via centrifugation (1700 rpm) for 10 min. The black product was dissolved in chloroform and centrifuged (3000 rpm) for 5 min to remove the precipitate, and the supernatant was stored at 4°C.

60 and 80 nm: 60 and 80 nm nanoparticles were synthesized according to the previous report.[2] Briefly, $Fe(acac)_3$ (2 mmol) was added to a mixture of oleic acid (1.13 g) and benzyl ether (10.4

g). The mixture solution was degassed at room temperature for 1 h and then heated to 290°C under vigorous magnetic stirring. The reaction mixture was maintained at this temperature for 30 min. The precipitated process was the same as that of 40 nm NPs.

Synthesis of VS-4718⊂L and Paclitaxel⊂L nanoparticles

VS-4718⊂L and Paclitaxel were added into the ethanol solution of a mixture (L-α-Phosphatidylcholine (PC, 3.04 mg), cholesterol (CH, 0.72 mg), and DSPE-PEG-NHS (0.3 mg). VS-4718⊂L and Paclitaxel⊂L were formed by rapidly injecting the ethanol solution into PBS (1 mL), followed by adding 0.02 mg peptide RGD, which increased the targeted ability of VS-4718⊂L and Paclitaxel⊂L to tumor cells, stirring for 2 h. The ethanol in the liposome solution was evaporated with a rotatory evaporator. Store the dialyzed nanoparticles at 4°C.

Characteristics of magnetic nanoparticles

The synthesized magnetic nanoparticles were characterized with a transmission electron microscope (TEM) and dynamic light scattering (DLS). The morphology and size of NPs were examined on a Tecnai G2 20 S-TWIN TEM at an acceleration voltage of 200 kV. DLS experiments were performed with a Zetasizer Nano instrument (Zetasizer Nano ZS) equipped with a 10-mW helium-neon laser (λ = 632.8 nm) and a thermoelectric temperature controller. The sample was dispersed in the PBS buffers (pH = 7.4) or chloroform. The aqueous phase transfer of hydrophobic magnetic nanoparticles was based on the previous report.[3]

Cell culture

Paclitaxel-resistant human lung cancer cells (Paclitaxel-resistant A549 cell lines) and Paclitaxel-sensitive cancer cells were incubated with DMEM medium supplemented with 10% FBS, 1% penicillin, and streptomycin at 37 °C in a humid environment and 5% CO_2.

Cytotoxicity

Nanoprobes: Cells (SMRCs and SCs) were seeded in 96-well plates at a density of $\sim 1\times 10^5$ cells per well and cultured further for 17 h. The different concentrations of nanoprobes (0, 5, 10, 20, and 50 μg/mL) were added to the medium, respectively. Then, the cell viabilities were investigated through CCK-8 cell counting kits after 36 h incubation.

VS-4718⊂L and Paclitaxel: The cells (SMRCs and SCs) were seeded in 96-well plates at a density of $\sim 1\times 10^5$ cells per well and cultured further for 17 h. The different concentration of Paclitaxel, VS-4718⊂L, or VS-4718⊂L and Paclitaxel⊂L was added into the medium, respectively. Then, the cell viabilities were investigated through CCK-8 cell counting kits after 36 h incubation.

The working profile of FIRMS

The working profile of FIRMS is that an external force with varying amplitudes is applied to the magnetically labeled samples, which interact with target molecules immobilized on the substrates and show a strong remanence signal after magnetization.[4] When the exerted force is equal to the interaction force between two molecules, the magnetically labeled samples will detach from their target molecules, resulting in a decrease of the overall remanence signal. This process is recorded by an atomic magnetometer.

The effect of nanoprobes on cell mechanical profiles

The cells were incubated with Paclitaxel (1 nM) and/or PBS for 36 h and then incubated with low and high concentrations of nanoprobes (25 and 50 μg/mL) in 6-well plates for 4 h. The cells were digested by trypsin (0.25%, 3 min) and re-seeded on the FIRMS slices to incubate for 30 min. After magnetization, the slice incubated with cells was examined by using FIRMS. The curve of the force-dependent relative magnetic signal was obtained to test cell mechanical profiles.

Determination of adhesion force, adhesion stability, and cell-cell force

The cells (SMRCs and SCs) were seeded in 6-well plates at a density of ~1×10^6 cells per well and cultured further for 17 h. After 4 h-incubation with nanoprobes (20 μg/mL), the Paclitaxel (1 nM) dispersed in BSA solution (2%) was added into the medium and cultured for 10 h or 36 h. Then the cells were digested with trypsin (0.25%) and reseeded on the special FIRMS slice coated with FN (26 μg/mL). Then measure the mechanical properties using FIRMS.

Adhesion force: According to the short-term adhesion assay,[5,6] the cells were incubated on the slice for 30 min. Initial magnetic signals of cells were detected after magnetization for 2 min. Then the centrifugal force was applied to the adhered cells, reducing cell detachment. FIRMS slice with cultured cells kept perpendicular to the centrifugal force and facing outward. The force-dependent cell detachment was recorded through the decreased magnetic signal.

Cell-cell force: Culture the treated cells for another 17 h, and then add the same number of cells to the medium, letting these fresh cells interact with the pre-seeded cells for 30 min. The cells were magnetized for 2 min, and then the centrifugal force was used to record the cell detachment through a decreased magnetic signal. The centrifugal force exerted on the cells was calculated according to the previous report[6]:

$$F = (\rho_{\mathrm{cell}} - \rho_{\mathrm{medium}}) \cdot V_{\mathrm{cell}} \cdot \omega^2 \cdot r$$

where F is the relative centrifugal force, ρ_{cell} and ρ_{medium} is the specific density of the cells (~1.07×10^3 kg/m^3) and the medium (1×10^3 kg/m^3), respectively. V_{cell} is the cell volume (~525 μm^3). r is the radius of the rotation (0.045 m). The relative adhesion force was defined as the force corresponding to the half-maximum signal. In the control experiment of integrin blocking,

after the cells were treated with Paclitaxel for 36 h, the cell medium was changed to the complete medium. The peptide (PLGVRGRGD, 5 mg/mL) synthesized according to our previous report[7] was added to the medium and cultured for 15 min at 37 ºC. Then the cells were digested to examine the mechanical behavior. In the control experiment of FN abrogating, the FIRMS slice was not modified with FN but just cultured with a BSA (1%) solution before utilization.

Adhesion stability: The cells were incubated on the slice for 30 min. A constant force (25 pN) was imposed on the cells for more than 6 repeats. The adhered cells were counted by microscopy. Measurement of contraction force via elastic micropillar system according to the manufacturer's instructions from Microduits Company (Switzerland).

Investigation of cell promigratory and migration

Promigratory: Cells (~1.5×10^5) were incubated on the FIRMS slices coated with FN. After the cells (SCs and SMRCs) were incubated on the slice for 17 h, the medium was changed with the complete DMEM medium containing Paclitaxel and incubated for 36 h. Nanoprobes (20 μg/mL) were added to the cell medium without FBS and incubated for 4 h, and then the medium was washed with PBS 3 times. The trypsin (0.25%) was added to the solution of the medium. The time-dependent signal decrease induced by cellular deformation and promigratory was monitored by FIRMS.

Migration: Cells were incubated on the FIRMS slice for 8 h with DMEM medium supplemented with 10% FBS, 1% penicillin, and streptomycin at 37 ºC in humidity and 5% CO_2 atmosphere. The fresh cell medium without FBS was changed to incubate cells with nanoprobes (20 μg/mL). After incubation for 4 h, the slice was washed 3 times with the medium. The cells were further incubated with Paclitaxel (1 nM) for 10 h and 36 h, then digested with trypsin (0.25%) and re-seeded on the FIRMS slice for 1 h. After magnetization, the migration-induced signal decrease was recorded by FIRMS.

Wound healing assay

Cells were seeded into 6-well plates. The migration velocity of treated cells was examined through a thin "wound" introduced by scratching with a pipette tip. Cells at the wound edge polarized and migrated into the wound space, and the migration rates were analyzed by wound space distance at 36 h.

Investigation of cell polarization via microscopy

Cells (SMRCs and SCs) were seeded in a PDMS slice at a density of ~1×10^6 cells and cultured further for 17 h. The Paclitaxel (1 nM) dispersed in BSA solution (2%) was added into the medium and cultured for 36 h. The cell polarization was observed by F-actin polymerization

through Actin-stain 555 phalloidin staining. The polarization ratio of the cells was calculated from the ratio of the significantly polarized cells (length: width > 2) to total cells in microscopy images.

Immunofluorescence staining

Cells were fixed and processed for immunofluorescence as described in previous reports.[8] Briefly, the treated cells were fixed in 4% paraformaldehyde for 15 min after washing 5 times with warmed PBS. All these processes were performed at 37 °C. After the fixation step, the cells were treated with 0.5% Triton X-100 (5 min) for permeabilization in DPBS at room temperature, and then the cells were washed three times with DPBS to remove the detergent. To enhance the specific interaction of the antibody to targeted proteins, non-specific antibody binding was blocked by cell incubation with 1% (w/v) BSA in PBST (0.1% v/v Tween 20) at room temperature for at least 1 h. The cells were then washed briefly with DPBS 3 times. F-actin staining: the working stock (100 nM) of Actin-stain 555 phalloidin (Sigma-Aldrich) diluted with DPBS (0.1% BSA) was added to the cells for incubation for 1 h. P-MLC II, vinculin, merlin, tubulin, P-, E-cadherin, and phosphorylated (pSer518) merlin staining: the primary antibody (Abcam, P-cadherin, ab242060; E-cadherin, ab1416; merlin, ab88957, P-MLC II, ab2480, vinculin ab129002, tubulin, ab6046) diluted in 1:300 ratio in DPBS with 0.1% BSA was added into the cells and incubation overnight at 4°C. After incubation and washing 3 times with DPBS, cells were incubated with a secondary antibody tagged with the fluorescent dye of Alexa Fluor 677 (Sigma-Aldrich) diluted in a 1:300 (v/v) ratio with DPBS (0.1% BSA) for 1 h at room temperature. The nuclei were stained with 4', 6-diamidino-2-phenylindole (DAPI) for 10 min at room temperature with washing for 5 min per time (5 times). The image series was acquired using a 100X phase objective lens of a confocal microscope (OLYMPUS FV1000-IX81) at 405 nm, 559 nm, and 633 nm of laser excitation. Fluorescence intensity was quantified using ImageJ software from a minimum of 10 randomly selected images per condition, with 20–50 cells analyzed per image. The data represent three independent experiments.

Young's modulus measurement using atomic force microscope (AFM)

AFM measurements were performed as previously described. Briefly, the cells were cultured in the cell culture dishes with a complete medium and placed on the stage for AFM measurements. AFM studies were conducted using a Dimension FastScan Bio Atomic Force Microscope (AFM, Bruker) with a combined inverted optical microscope. The measurements were collected at 37 °C using silicon nitride cantilevers with spring constants of 0.07 N/m and a tip radius of 20 nm (MLCT-BIO-DC, Bioscope catalyst AFM from Bruker). The scan size for all measurements was set to 0 nm to maintain a constant position over the cell. The AFM tip

was brought into contact with the central region of the cell. The Young's modulus was identified using a modified Hertz model, and the data were analyzed using Nanoscope software.

Micro-fabricated elastic micropillar assay

The micropillar devices were purchased from MicroDuits (www.microduits.com). The assay was run according manufacturer's instructions, and the image was analyzed with their Open Source Software "MechProfiler". Briefly, the glass substrate with micro post array was put in a 12-well plate and wet by adding 1 mL ethanol (99%) and incubated at room temperature for 20-30 s. Then 1 mL DI water was added to dilute the ethanol, and then 1 mL was aspirated away, and this step was repeated at least 3 times. Replace the DI-water with PBS in the same manner, and then 1 mL was also aspirated away. Repeat this step 3 times. After these steps, the PBS was replaced by adding 1 mL of cell culture medium and then taking out 1 mL of medium. This step was also repeated at least 3 times. Then the 1-mL cell solution (250000 cells) was added on top of the micropost array and cultured in an incubator (CO_2, 5%; 37 °C) for 6-7 h. The cells were fixed with 3.7% formaldehyde solution for 5 minutes, and then the cells were stained with dye (0.05% Coomassie Brilliant Blue in 50% water, 40% ethanol, and 10% acetic acid) for 90 s. The micro post arrays were observed using the 20X objective of the Confocal with the micro post array in the imaging dish, with the micro posts facing up. After obtaining the images with micro post tips in focus, the MechProfiler software was used to analyze images under the guidance of the manufacturer.

Cell labeling assay

MRCs and SCs were seeded in 6-well plates at a density of ~2×10^6 cells per well and cultured further for 17 h, respectively. DiI and DiO (6 μM) were added to the medium of MRCs and SCs, respectively, and cultured for 5 min. The cells were digested using trypsin (0.25%) for 3 min at 37 ºC. The labeled cells (MRCs, 0.5%, and SCs, 99.5%) were mixed to form SMRCs, then reseeded in confocal plates for 17 h and treated with Paclitaxel (1 nM) for 10 h or 36 h. The cell's behavior was observed by using a confocal microscope (OLYMPUS FV1000-IX81) at 488 nm and 555 nm of laser excitation.

Conditioned medium culture

Paclitaxel-resistant cells (~1×10^5) were seeded in the 96-well plates, and the media were conditioned for 36 h by culturing Paclitaxel-resistant cells with Paclitaxel (1 nM). Then the supernatant (200 μL) collected as a conditioned medium was added to the SCs (~1×10^5) and incubated in 96-well plates. The adhesion force and migration rate of SCs incubated with a conditioned medium were examined as the above methods.

3D tumor spheroid culture

5.0×10^4 cells (SCs or SMRCs) were seeded in 400 μL assay medium (DMEM, 10% FBS, 1% penicillin and streptomycin, and 2% Matrigel) in 6-well chamber slides with 60 μL of Matrigel matrix (BD). Tumor spheroids were formed and cultured for 15 days. The medium (DMEM, 10% FBS, 1% penicillin and streptomycin, and 2% matrigel) was exchanged for 4 days.

Cell invasion in 3D culture system: At 15 days, the two groups of tumor spheroids (SCs and SMRCs) were treated with Paclitaxel (1 nM). The other two groups were treated with the same volume of PBS. Finally, the invading cells were imaged by a microscope with a 10× lens.

Cell viability in 3D culture system: After 15 days, the tumor spheroid was treated with PBS, Paclitaxel⊂L (10 nM), VS-4718⊂L (10 μM), Paclitaxel⊂L (10 nM) +VS-4718⊂L (10 μM), respectively, for 24 h. The cell viability was measured using the CCK-8 kits. The tumor spheroids were observed using live/dead cell staining kits.

Transwell migration and invasion assays

12-well cell migration assay kits with 8-μm pores were obtained from BD Company. Serum-starved cells were seeded in the upper chamber and allowed to migrate for 36 h into DMEM/10% FBS in the lower chamber. The invasiveness of all cell lines was assayed by migration through a commercially available 12-well invasion assay (BD BioCoat Matrigel Invasion Chamber). As per the manufacturer's instructions, Matrigel inserts on an 8 μm pore-size filter were rehydrated with warm DMEM for 2 h, and then serum-starved cells were added to the upper chamber and allowed to invade for 36 h into DMEM containing 10% FBS. Cells that passed through were detached from the membrane and quantified using the CCK-8 kits supplied in a Microplate reader with 450 filter sets.

Microfluidic assay

The microfluidic device was prepared by PDMS with a microchamber ($0.5 \times 4 \times 4.5$ mm^3). The micro-chamber surface was modified with FN. The PDMS microfluidic channel was incubated with DMEM/10% FBS for at least 1 h. Then, the pre-treated cells were injected into a microfluidic channel under different shear stresses by adjusting the fluid velocity (50, 100, 150, 250 μL/min) for 30 min. The adhered cell number was recorded through microscopy. The sheer force of the fluid was calculated through the method reported in the previous study.[5]

***In vivo* invasion study**

Cells (SMRCs and SCs) xenografted tumors were inoculated into the right flank of the 6 to 8-week-old female BALB/c nude mice. When the tumors had developed to about 4 mm in diameter, the mice were randomly divided into four groups (n=6 mice per group) in a way to minimize weight and tumor size differences among the groups and administered with PBS and Paclitaxel in BSA (0.2 mg/mL) (200 μL, pH 7.4). The drug was intravascularly injected once a

time per five days (d = 0, 5, 10, 15, 20). On 25 days, the tumor cells were resected and isolated according to the previous reports. The tumor cells were digested by 1% trypsin in 10 mL HBSS solution containing $CaCl_2$ (1 M) for 1 h at 37 °C. After getting the cell suspension, they were filtered through a sterile filter (70 μm, BD) and centrifuged (1000 rpm) for 5 min. The tumor cells were resuspended in DMEM complete medium and further cultured for 4 generations. Then, the metastasis contribution factors (mechanical force and migration) were investigated with FIRMS and microscopy. All experiments were performed three times.

***In vivo* metastasis experiments**

Six-week-old female BALB/c nude mice were injected with 2.0×10^5 cells in 100 μL of PBS+10% OptiMem into the tail vein on day 0. Mice were injected with each cell line, for 6 mice per group. On day 25, all animals were euthanized with CO_2. Organs (Lungs, hearts, kidneys, livers, and spleens) were then removed and fixed in 15 mL of 10% neutral-buffered formalin overnight. After fixation for 16 h, metastases were counted, and tissue was imaged. The metastasis ratio was calculated from the metastatic mice divided by the total mice per group.

Tumor therapy

SMRCs were inoculated into the right flank of the 6 to 8-week-old female BALB/c nude mice. When the tumors had developed to about 4 mm in diameter, the mice were randomly divided into four groups (n=5 mice per group) in a way to minimize weight and tumor size differences among the groups. The mice were administered with PBS, Paclitaxel⊂L (0.1 mg/mL, 200 μL), VS-4718⊂L (1 mg/mL), Paclitaxel⊂L+VS-4718⊂L, respectively, through tail vein injection. The drug was injected once a time per day on days 11, 13, 15, 17, and 19.

Tissue histology and immunohistochemistry

Formalin-fixed and paraffin-embedded mouse xenografts primary tumors, and organ tissues (lung, heart, liver, spleen, and kidney) were sectioned and stained with H&E and Picrosirius Red. For immunohistochemistry, the slice was stained overnight at 4 °C with antibodies against collagen I (Abcam), followed by incubation with Alexa Fluor 674 tagged secondary antibody and Actin-stain 555 phalloidin (Beyotime Biotechnology, China) for 2 h at room temperature.

RNA sequence experiments.

The SMRCs and SCs were cultured for 48 h, and then the cells were transferred into 1.5 mL RNAase-free tubes and stored in liquid nitrogen. The RNA extraction and sequence analysis were performed by the company Novogene.

Reverse Transcription-Quantitative Polymerase Chain Reaction (RT-qPCR).

For measurement of related mRNA levels, total RNA of cells (SCs or SMRCs) was isolated via RNA Easy Fast Tissue/Cell Kit (Tiangen Biotech, Cat# DP451) according to the vendor's

recommendations. Then, one hundred nanograms of RNA was converted to complementary DNA (cDNA) via ReverTra Ace qPCR RT Master Mix with gDNA remover (Toyobo Co., Cat# FSQ-301S). The expressions of Vimentin mRNA (the forward primer: 5'-AGATGGCCCTTGACATTGAG-3'; the reverse primer: 5'-TGGAAGAGGCAGAGAAATCC-3') and E-Cadherin mRNA (the forward primer: 5'-GAAGTGTCCGAGGACTTTGG-3'; the reverse primer: 5'-CAGTGTCTCTCCAAATCCGATA-3') were quantified with a Bio-Rad iQTM5 real-time PCR detection system. The mRNA levels of target genes were normalized to the gene expression of endogenous control GAPDH.

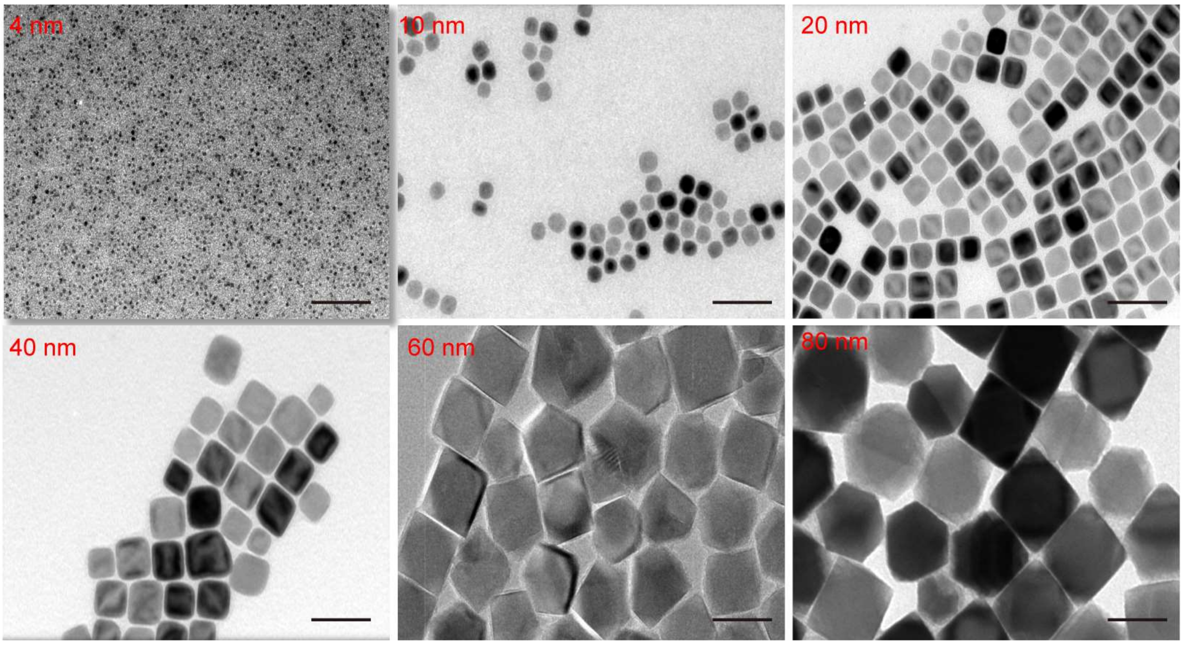

Figure S1. Morphology and size of magnetic nanoparticles with different sizes from 4 nm to 80 nm. Scale bar: 50 nm.

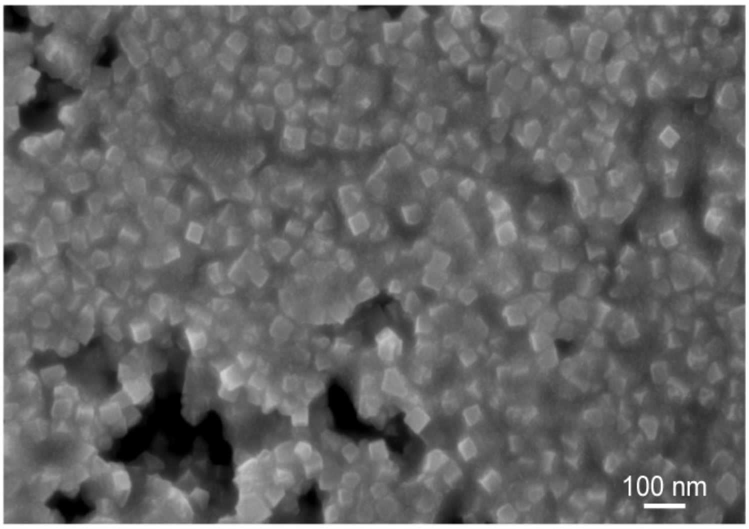

Figure S2. Scanning electron microscopy (SEM) image of magnetic nanoparticles used in this work.

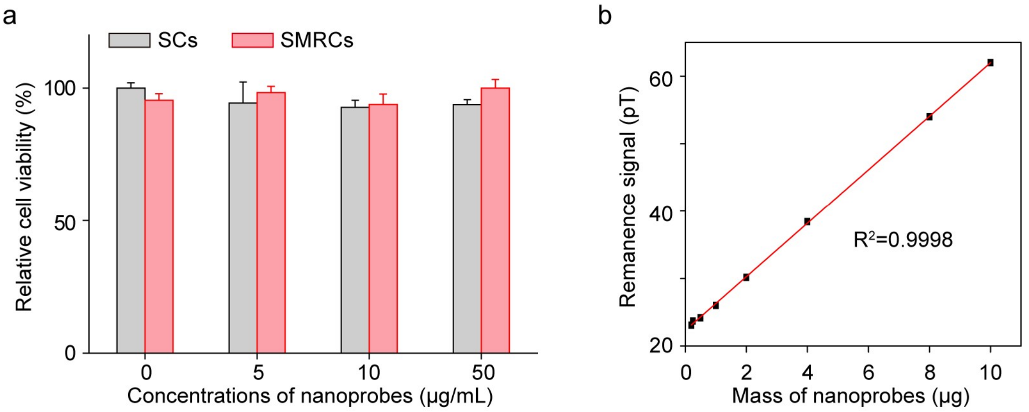

Figure S3. a) Effect of nanoprobes on cell viability. Cells were treated with different concentrations of nanoprobes (0, 5, 10, and 50 μg/mL) for 36 h, respectively. b) Linear relationship between magnetic signal and the mass of nanoprobes.

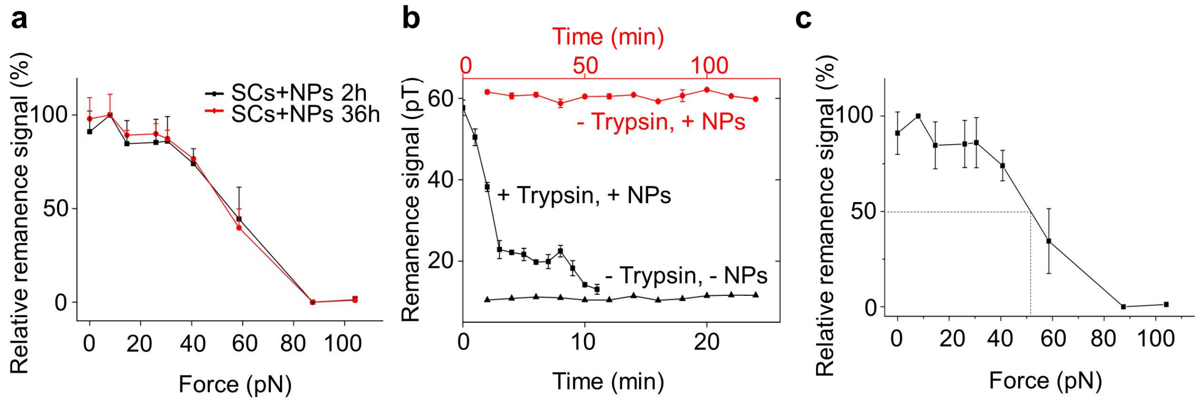

Figure S4. a) Curves of force-induced remanence signal decrease for evaluating the effect of nanoprobes on adhesion force. b) Curves of remanence signal changes imposed by the external mimic force (0.25%, trypsin) for evaluating the sensitivity of FIRMS on the measurement of deformation, migration, and detachment of nanoprobe-labeled cells. c) Curves of force-dependent relative signal decrease of A549 cells recorded by FIRMS to measure the cell adhesion force. Data are mean ± SD, n = 3 independent experiments.

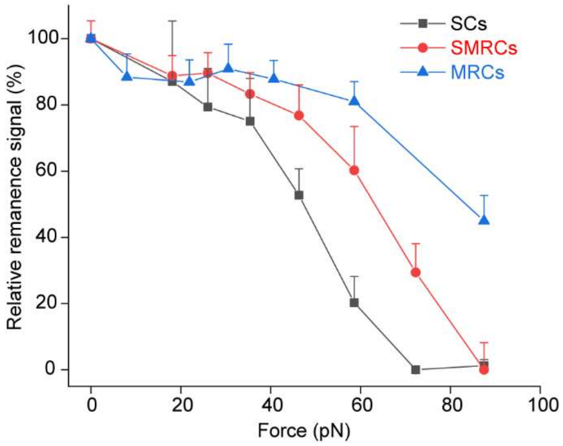

Figure S5. Curves of force-dependent relative signal decrease of SCs, SMRCs and MRCs. Data are mean ± SD, n = 3 independent experiments.

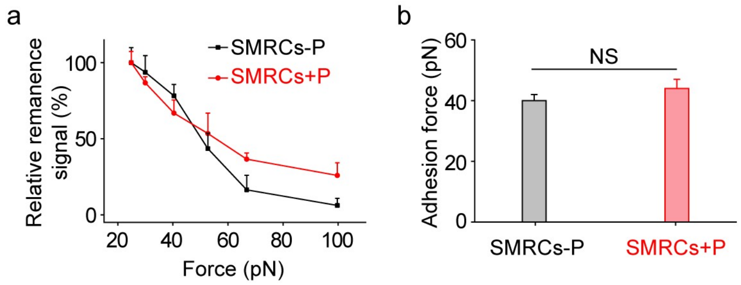

Figure S6. Curves of force-dependent relative signal decrease (a) and quantification of cell adhesion forces (b) of SMRCs when the mechanical properties of MRCs were inhibited by blebbistatin for 2 h (+B, 50 µM). Data are mean ± SD.

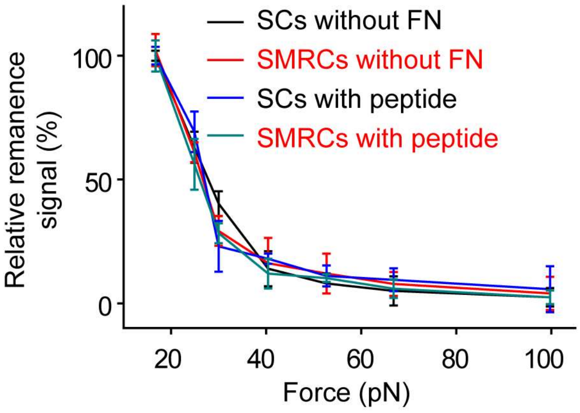

Figure S7. Adhesion force of the cells cultured on the substrates without FN modification and the cells treated with RGD-peptide.

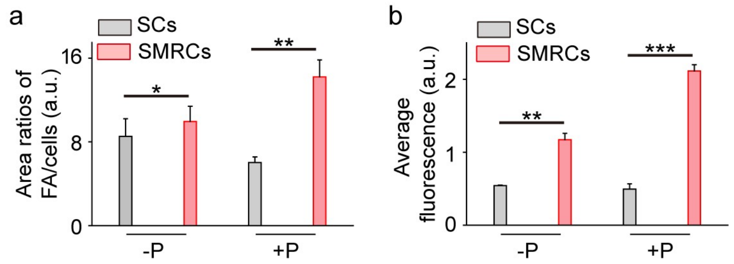

Figure S8. a) and b) Quantification of area ratios of FAs to cells (a) and average fluorescence intensity of FAs in cells (b) under different treatments. The fluorescence was quantified using Image J. Data are mean ± SD, *$P < 0.05$, **$P < 0.01$, ***$P < 0.001$.

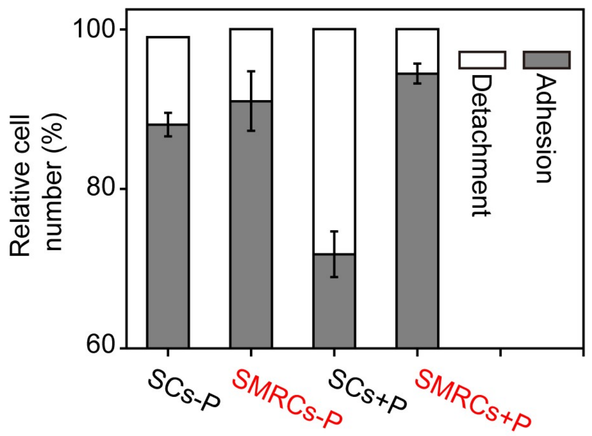

Figure S9. The percentage of adhered-to detached cells after applying force the sixth time. Force = 25 pN.

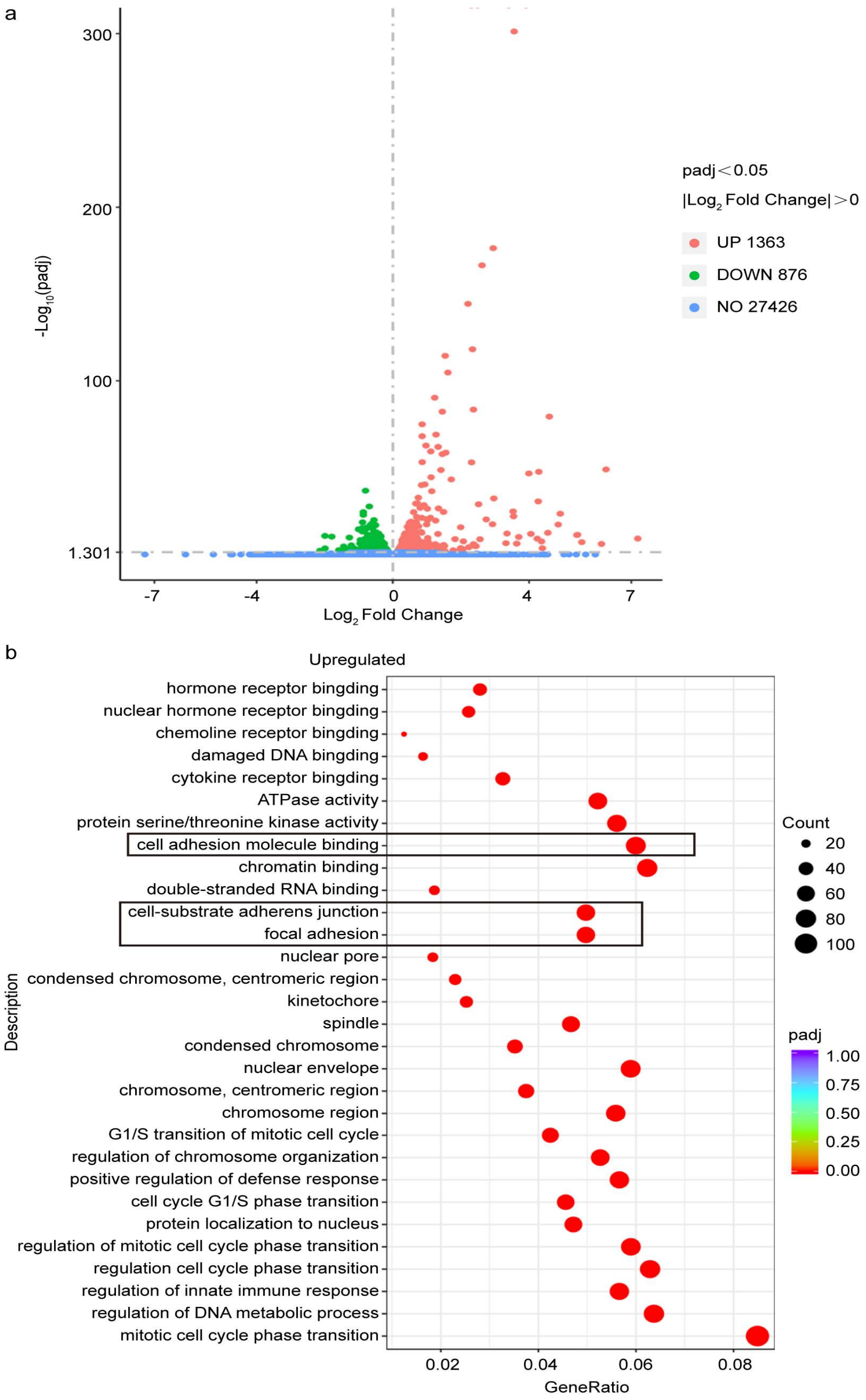

Figure S10. a) Volcano plot showing $\log_{10}$(adj) versus $\log_2$ fold change showed a summary of significant up-and-down-regulated genes between SMRCs and SCs. b) Go-term enrichment analysis based on the RNA-sequence data of the up-regulated genes in SMRCs versus SCs.

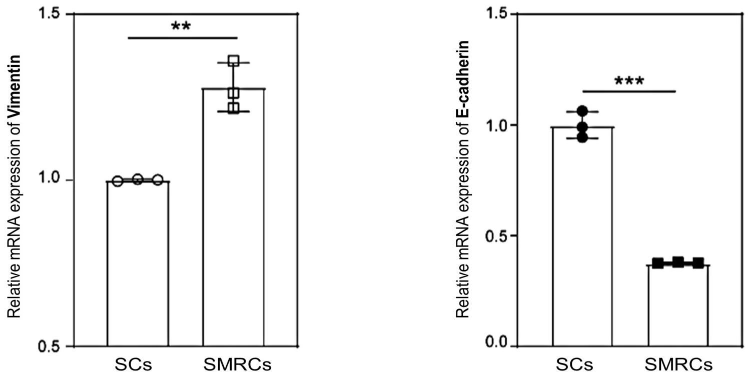

Figure S11. Relative expression levels of Vimentin and E-cadherin were quantified from RT-qPCR and normalized to GAPDH mRNA. Data are represented as mean ± SD (n = 3). $^{**}P < 0.01$, $^{***}P < 0.001$. Vimentin acts as a mesenchymal marker, and its levels generally rise during the epithelial-mesenchymal transition (EMT). This increase can make tumor cells less responsive to drug treatments. E-cadherin plays a crucial role in maintaining the adhesion between epithelial cells. When E-Cadherin expression is low, it can promote the EMT process and reduce the chemotherapy sensitivity of tumor cells.

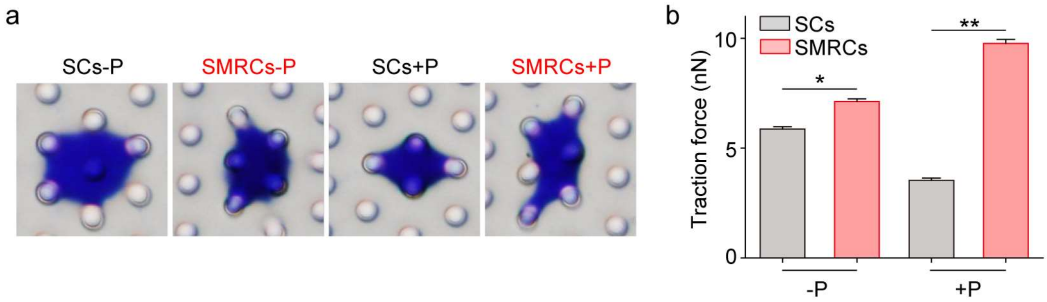

Figure S12. a) and b) Representative Trypan blue-dye-stained cells on FN-fabricated elastic micropillar (a) and quantifications of average adhesion forces exerted by cells under different treatments (b). Measured through ~200 cells per replicate. $^{*}P < 0.05$, $^{**}P < 0.01$.

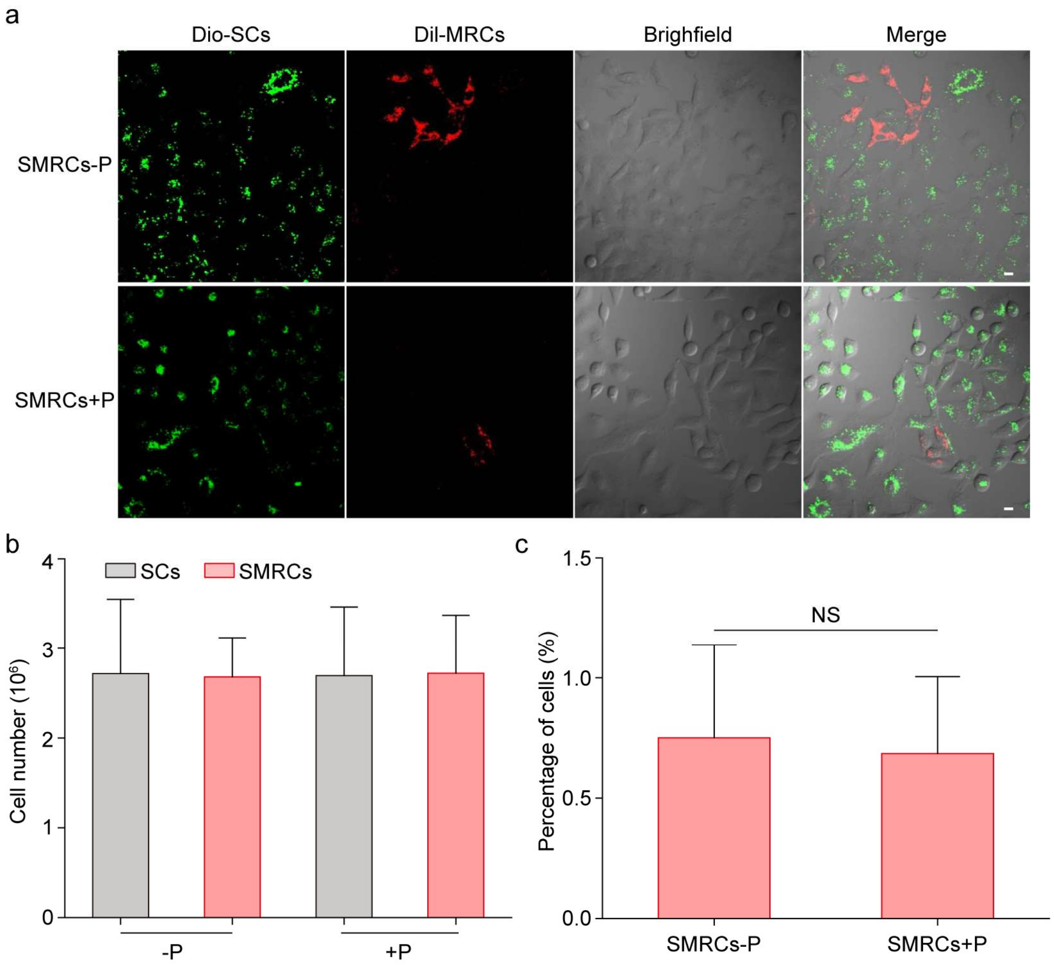

Figure S13. a) Representative subpopulation in SMRCs after 36 h incubation. Scale bar: 10 μm. Before co-culturing, MRCs and SCs were stained with DiI and DiO for 5 min at 37 ºC, respectively. n = 6 replicates. b) Total cell number of SCs and SMRCs after 36 h incubation under different treatments. Data are mean ± SD, n = 6 replicates. c) Percentage of MRCs in SMRCs with or without Paclitaxel treatment after 36 h incubation. Data are mean ± SD, NS, no significant difference, n = 6 replicates.

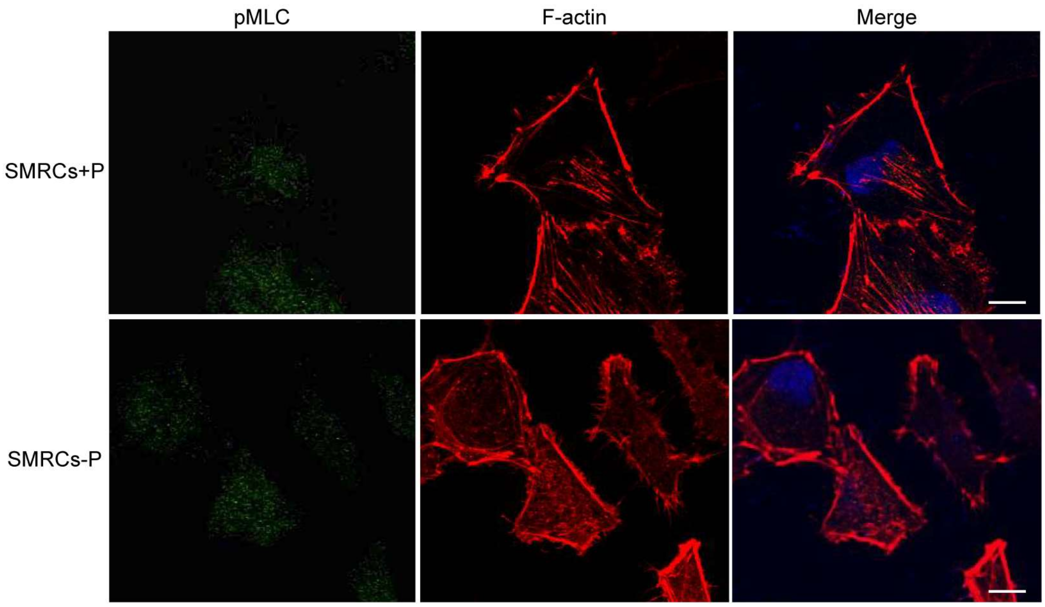

Figure S14. Representative confocal images of F-actin and pMLC in SMRCs with and without Paclitaxel by inhibiting the contractility of MRCs by adding the myosin inhibitor, blebbistatin (+B, 50 μM), F-actin (Red), pMLC (Green), and nuclei (Blue). Scale bar: 10 μm.

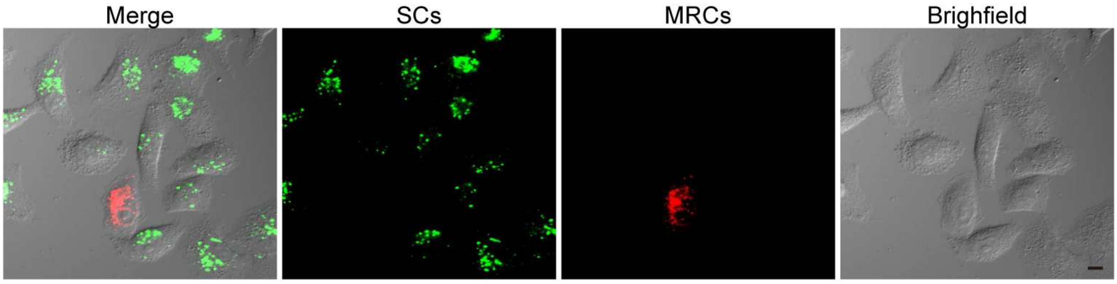

Figure S15. The polarization of MRCs as leader cells was disturbed by inhibiting the mechanical properties of MRCs before co-culturing. Before co-incubation, MRCs and SCs were stained with DiI and DiO, respectively, for 5 min at 37 °C. Scale bar: 10 μm.

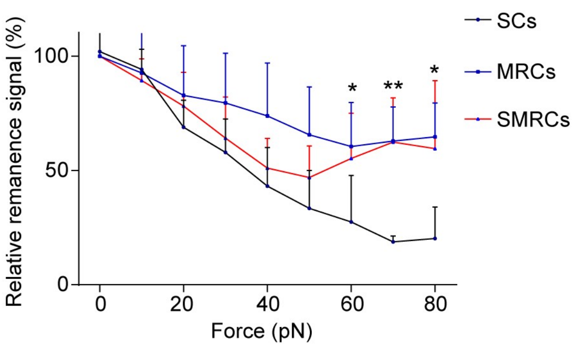

Figure S16. The cell-cell interaction force among SCs, MRCs, and SMRCs measured by using FIRMS.

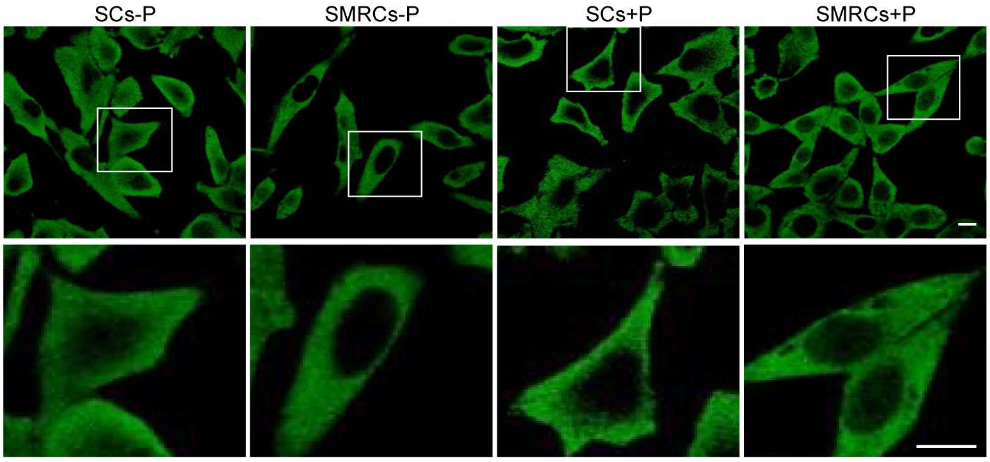

Figure S17. Representative immunofluorescence images of phosphorylated (pSer 518) merlin. The images shown at the bottom were the region of dashed squares in the corresponding top images. Scale bar: 10 μm.

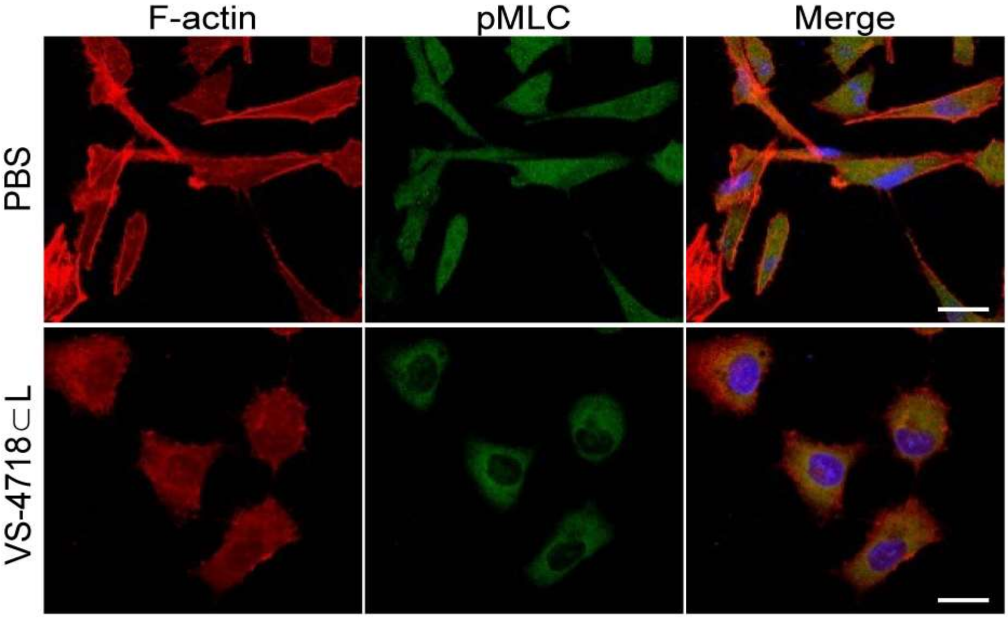

Figure S18. Confocal images of F-actin and pMLC in SMRC cells under different treatments (PBS and VS-4718⊂L). Staining for F-actin (Red), pMLC (Green), and nuclei (Blue). The contraction (F-actin and P-MLC) of SMRCs was decreased by VS-4718⊂L. Scale bar: 20 μm.

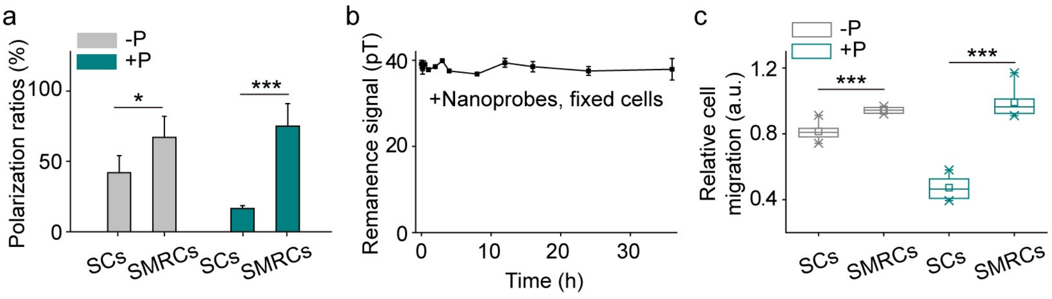

Figure S19. a) Polarization ratio of cells through statistical analysis of the protrusions spreading out distinctly in one or two directions. n ⩾ 300 cells. b) The stability of remanence signal in fixed cells. c) Cell migration rates obtained from FIRMS after 36 h treatment. Data are shown as the mean ± SD, $*P < 0.05$, $***P < 0.001$.

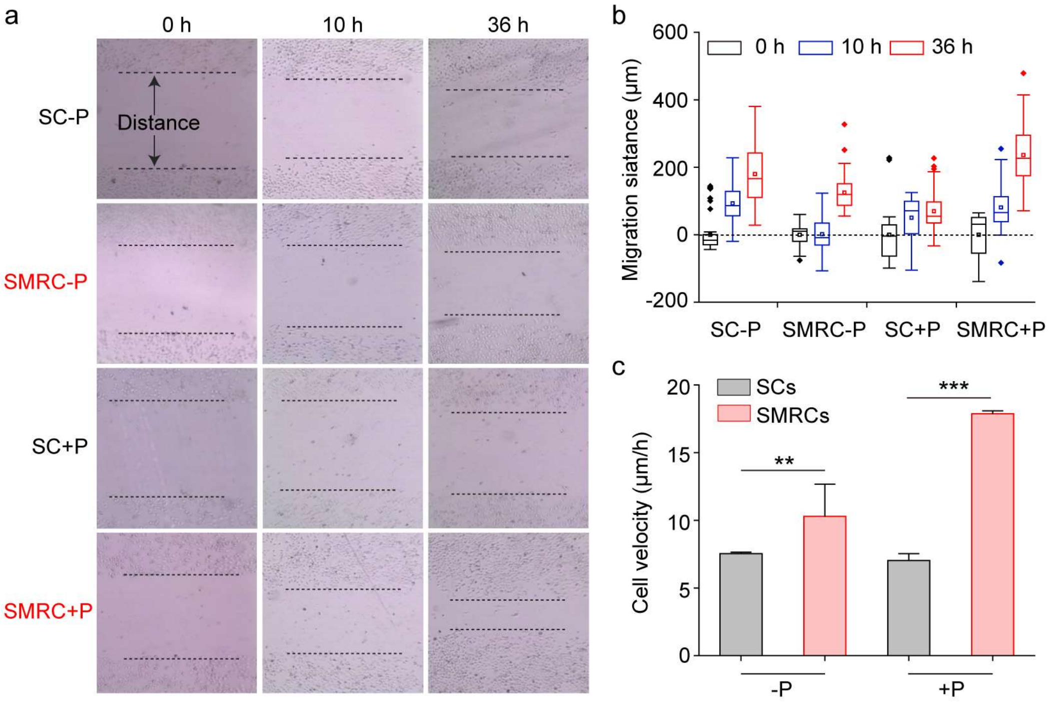

Figure S20. a) Representative images of cells migration across of wound space recorded by microscopy. Cells were incubated on the 6-well plates and treated with Paclitaxel or PBS for 10 h and 36 h, respectively. Then, a thin wound was introduced by scratching with a pipette tip in the middle of the plates. Cells at the wound edge migrated into the wound space. b) Statistical results of cell migration distance based on microscopy records. n = 6 replicates. c) Statistical results of cell migration rates based on microscopy records from the results of the wound healing assay. Data are shown as the mean ± SD, $^{**}P < 0.01$, $^{***}P < 0.001$.

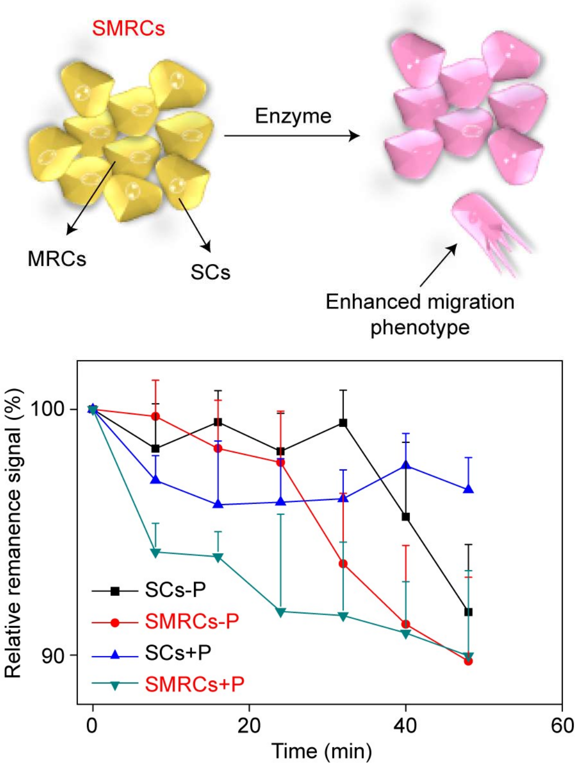

Figure S21. (Up) Schematic illustration of the individual cell in SMRCs obtaining a pre-migratory phenotype upon exposure to the digestive enzyme. (Down) Curves of time-dependent signal decrease recorded by FIRMS after cells were exposed to trypsin (37 °C, 0.25%). Enzyme-induced cell deformation or migration resulted in a decrease in remanence signal. n = 6 replicates.

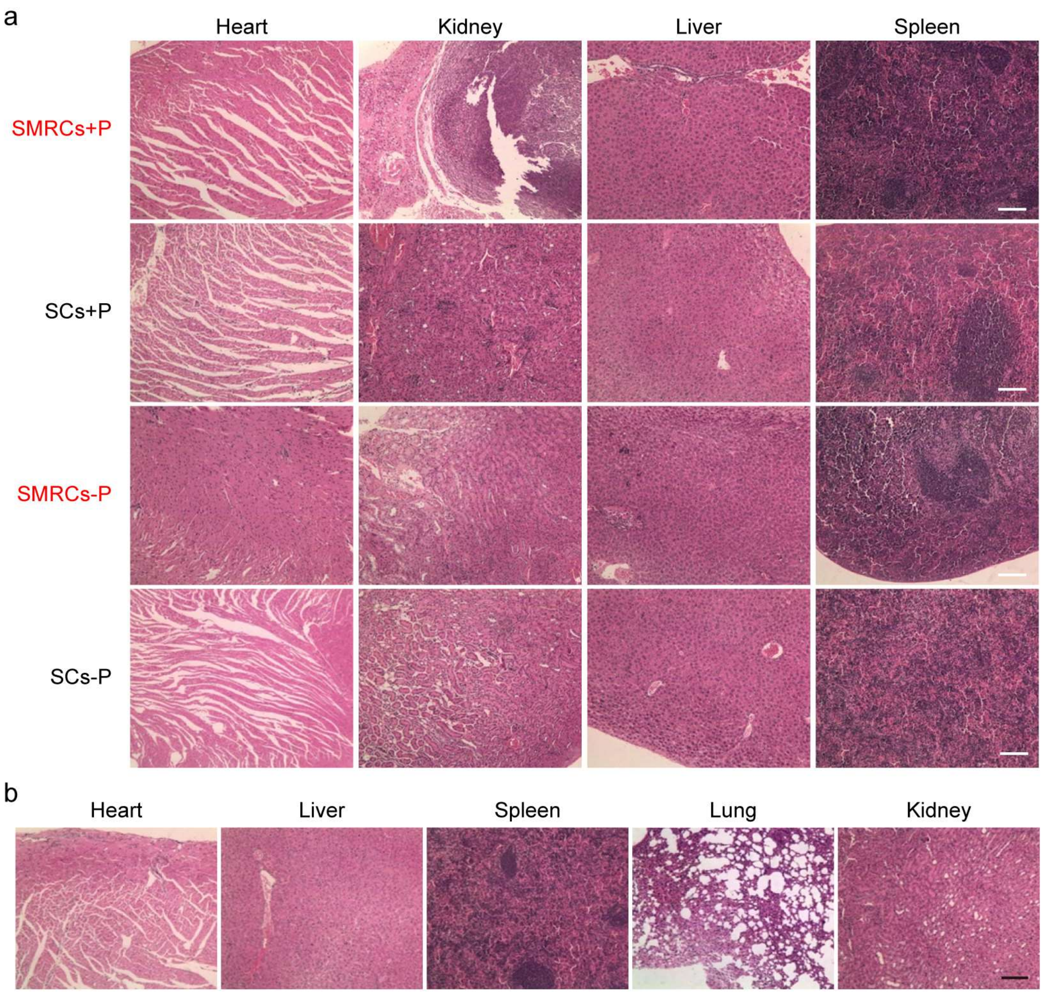

Figure S22. a) Representative image of H&E staining of tissues in different organs from mice subjected to tail vein injection with SCs and SMRCs, respectively. Scale bar: 500 μm. b) Representative images of H&E staining of tissues in different organs from mice subjected to tail vein injection with SMRCs in which MRC mechanical properties were inhibited by blebbistatin (50 μM) before co-incubation. Scale bar: 500 μm.

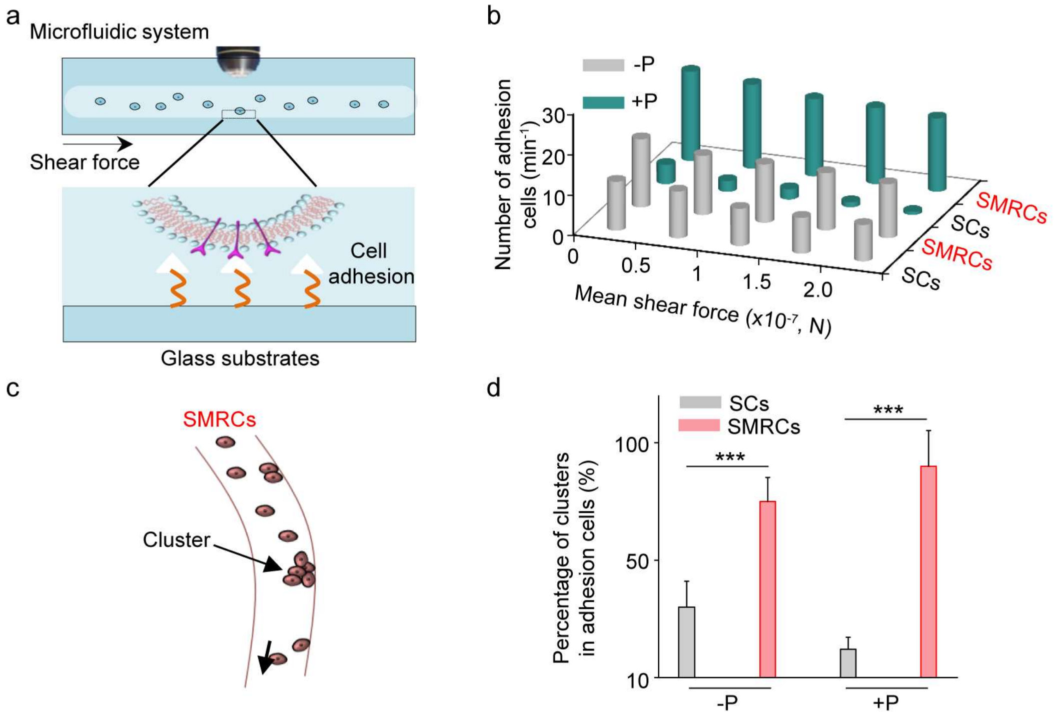

Figure S23. a) Schematic illustration of a microfluidic device combined with microscopy, which was utilized to mimic the vascular system. The microfluidic substrate modified with FN for arresting cells by integrin-based adhesion. b) The number of adhered cells under different shear forces in microfluidic substrates. c) Schematic illustration of SMRC-cluster formation in a microfluidic channel. d) Percentage of clusters in the adhered cells in microfluidic substrates recorded by microscopy. ***$P < 0.001$.

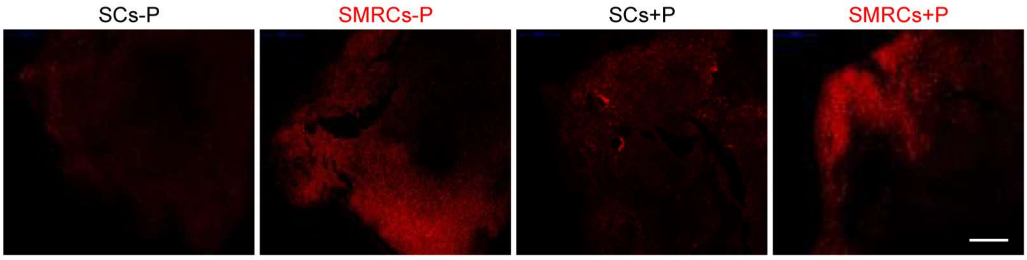

Figure S24. The tumor tissues of mice were stained with phalloidin. Scale bar: 500 μm.

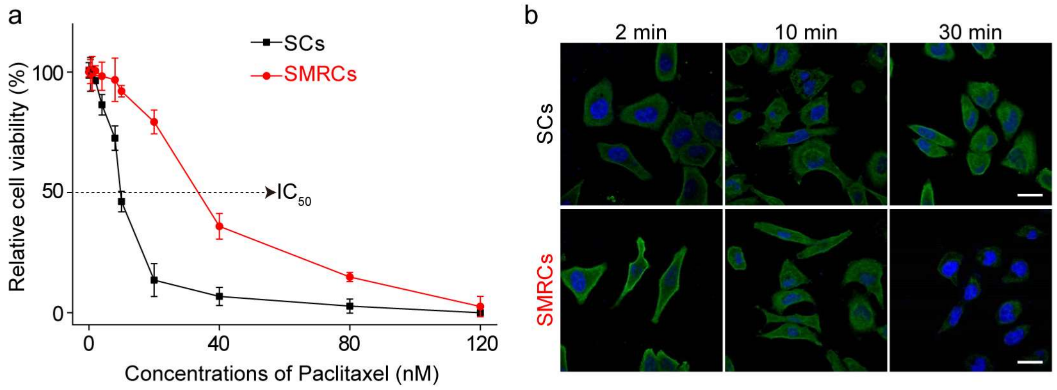

Figure S25. a) Relative cell viability of cells (SCs and SMRCs) cultured with different concentrations of Paclitaxel for 24 h. n = 3 replicates. Data are mean ± SD. b) Representative the dynamics of microtubules in the SCs and SMRCs. Scale bar: 20 μm. Tubulin (green) and nuclei (blue).

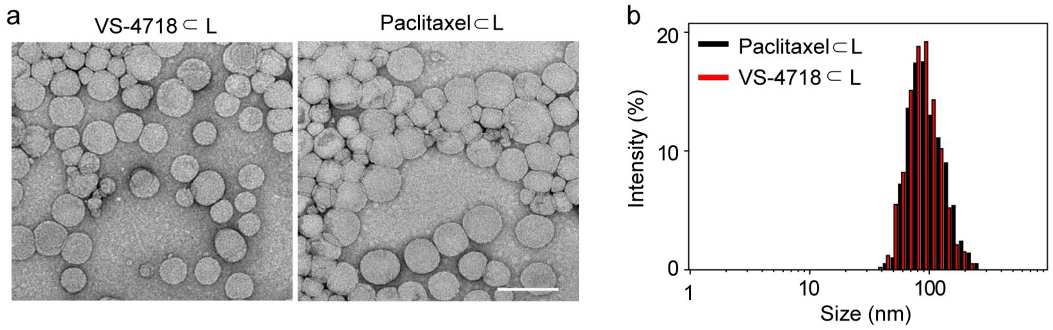

Figure S26. TEM images (a) and the hydrodynamic size distribution (b) of Paclitaxel⊂L and VS-4718⊂L nanovesicles. Scale bar: 200 nm.

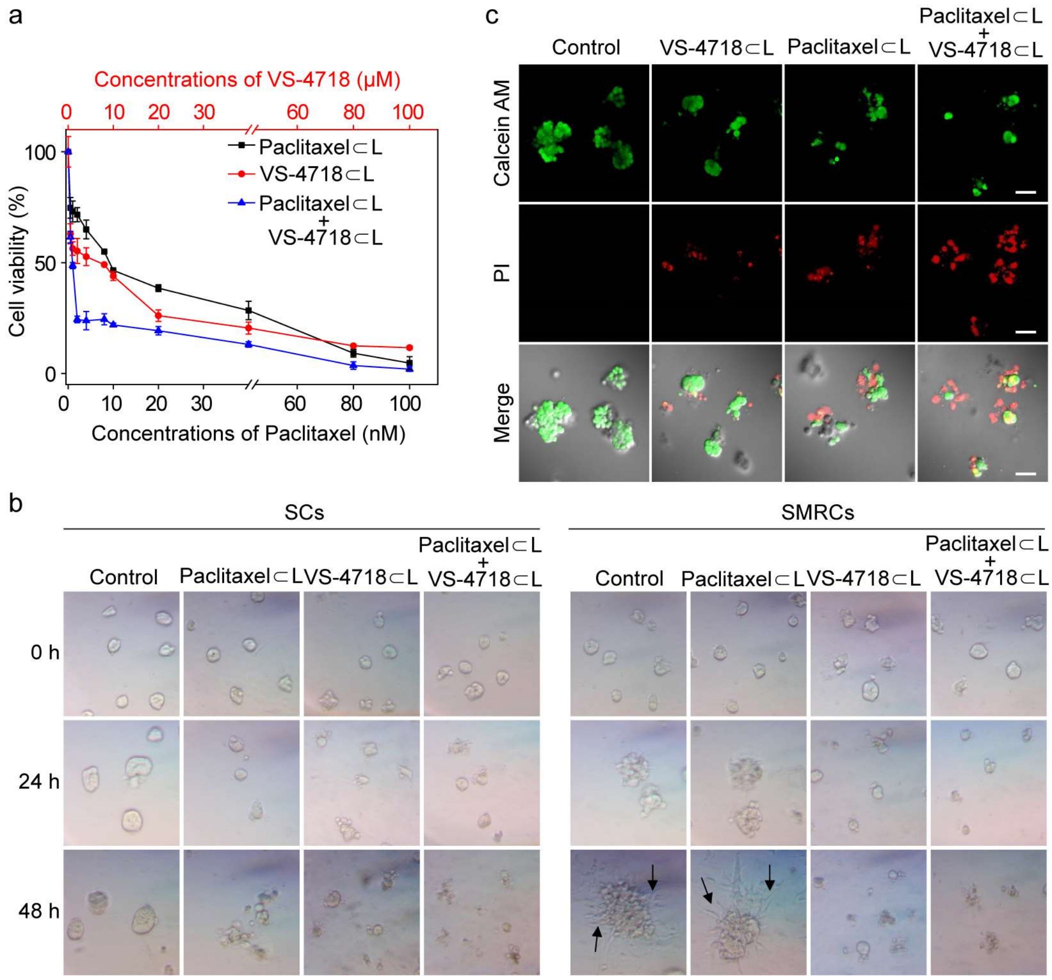

Figure S27. a) Relative cell viability of SC cells after treatments with Paclitaxel⊂L, VS-4718⊂L, together with Paclitaxel⊂L+VS-4718⊂L, compared with PBS. b) Microscopy images of time-dependent 3D tumor sphere (SCs and SMRCS) growth under different treatments. Arrow in SMRC tumor sphere treated with PBS or Paclitaxel⊂L represented the invading cells from the tumor sphere to the surrounding environment. c) Confocal images of the SC-tumor sphere growth and activity, detected through the live/dead cell staining. Scar bar: 200 μm.

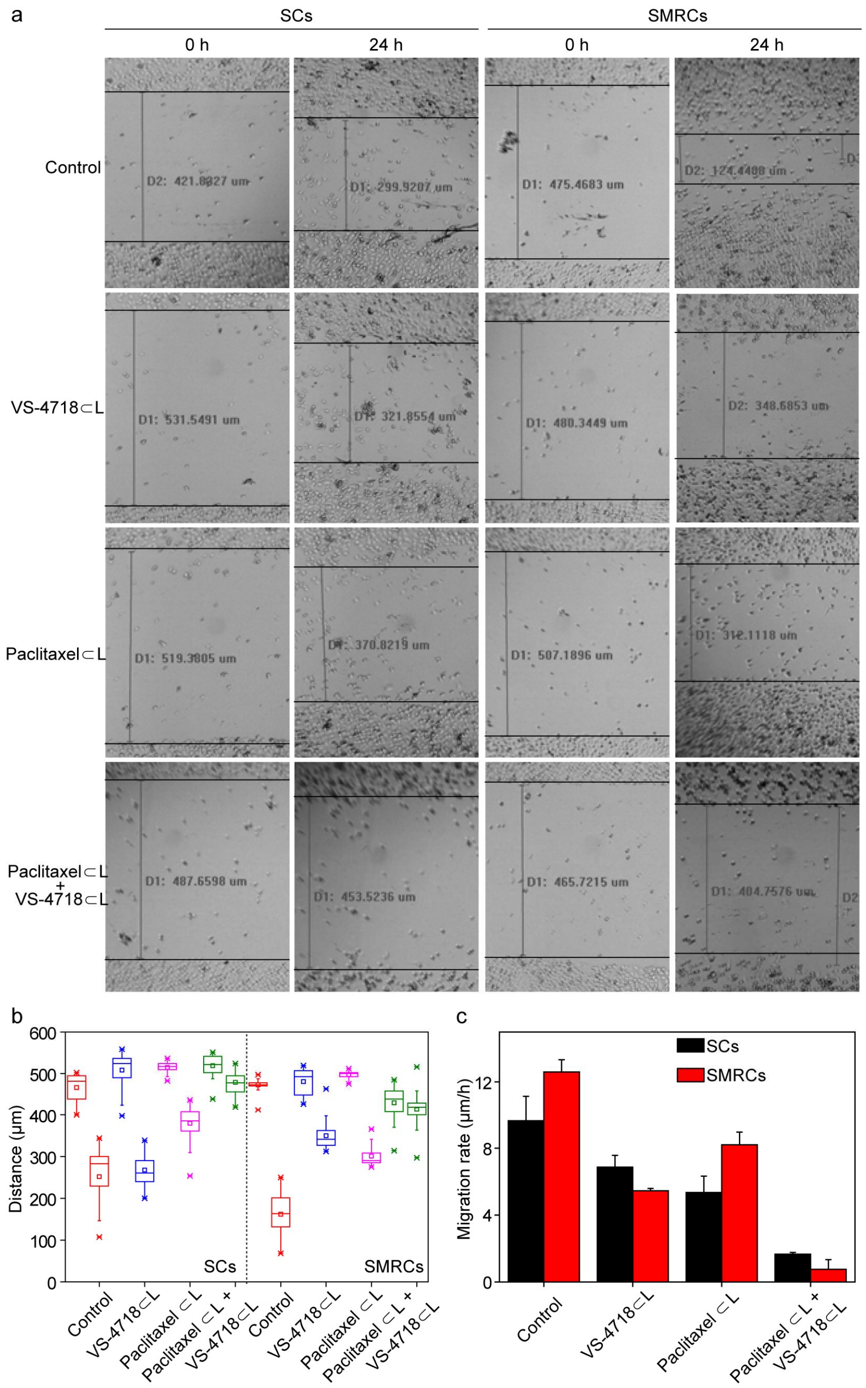

Figure S28. a) Representative images of cell migration across of wound space recorded by microscopy. Cells were incubated on the 6-well plates and a thin wound was introduced by scratching with a pipette tip in the middle of the plates, then the cells were treated with PBS, Paclitaxel⊂L, VS-4718⊂L, or together with Paclitaxel⊂L+VS-4718⊂L for 24 h. Cells at the wound edge migrated into the wound space. b) Statistical results of cell migration distance based on microscopy records. The two statistical boxes under each group were the wound width before (first) and after (second) migration. c) Statistical results of cell migration rates based on microscopy records from the results of wound healing assay. Data are shown as the mean ± SD.

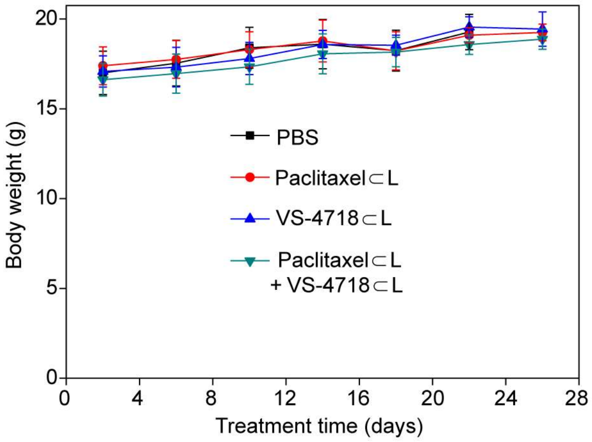

Figure S29. Body weight of mice with different treatments.

References

[1] Sun, S., Zeng, H., Robinson, D. B., Raoux, S., Rice, P. M., Wang, S. X., Li, G., Monodisperse MFe_2O_4 (M = Fe, Co, Mn) Nanoparticles. **2004**, *J. Am. Chem. Soc.*, *126*, 273.

[2] Kim, D., Lee, N., Park, M., Kim, B. H., An, K., Hyeon, T., Synthesis of Uniform Ferrimagnetic Magnetite Nanocubes. **2009**, *J. Am. Chem. Soc.*, *131*, 454.

[3] Liu, Y., Chen, T., Wu, C., Qiu, L., Hu, R., Li, J., Cansiz, S., et al., Facile Surface Functionalization of Hydrophobic Magnetic Nanoparticles. **2014**, *J. Am. Chem. Soc.*, *136*, 12552.

[4] Yao, L., Xu, S., Force-Induced Remnant Magnetization Spectroscopy for Specific Magnetic Imaging of Molecules. **2011**, *Angew. Chem. Int. Ed.*, *50*, 4407.

[5] Palecek, S. P., Loftus, J. C., Ginsberg, M. H., Lauffenburger, D. A., Horwitz, A. F., Integrin-Ligand Binding Properties Govern Cell Migration Speed through Cell-Substratum Adhesiveness. **1997**, *Nature*, *385*, 537.

[6] Reyes, C. D., García, A. J., A Centrifugation Cell Adhesion Assay for High-Throughput Screening of Biomaterial Surfaces. **2003**, *J. Biomed. Mater. Res. A*, *67A*, 328.

[7] Zhang, D., Qi, G.-B., Zhao, Y.-X., Qiao, S.-L., Yang, C., Wang, H., In Situ Formation of Nanofibers from Purpurin18-Peptide Conjugates and the Assembly Induced Retention Effect in Tumor Sites. **2015**, *Adv. Mater.*, *27*, 6125.

[8] Gupton, S. L., Waterman-Storer, C. M., Spatiotemporal Feedback between Actomyosin and Focal-Adhesion Systems Optimizes Rapid Cell Migration. **2006**, *Cell*, *125*, 1361.
